# Phosphorylation of IRE1 at S729 regulates RIDD in B cells and antibody production after immunization

**DOI:** 10.1083/jcb.201709137

**Published:** 2018-05-07

**Authors:** Chih-Hang Anthony Tang, Shiun Chang, Adrienne W. Paton, James C. Paton, Dmitry I. Gabrilovich, Hidde L. Ploegh, Juan R. Del Valle, Chih-Chi Andrew Hu

**Affiliations:** 1 The Wistar Institute, Philadelphia, PA; 2 Department of Molecular and Cellular Biology, Research Centre for Infectious Diseases, University of Adelaide, Adelaide, Australia; 3 Program in Cellular and Molecular Medicine, Boston Children’s Hospital, Boston, MA; 4 Department of Chemistry, University of South Florida, Tampa, FL

## Abstract

To relieve endoplasmic reticulum (ER) stress, IRE1 splices XBP1 messenger RNA (mRNA) or engages regulated IRE1-dependent decay (RIDD) of other mRNAs. Upon XBP1 deficiency, IRE1 switches to perform RIDD. We examined IRE1 in XBP1-deficient B cells and discovered that IRE1 undergoes phosphorylation at S729. We generated an anti–phospho-S729 antibody to investigate such phosphorylation. Compared with pharmacological ER stress inducers or Toll-like receptor ligands, the bacterial subtilase cytotoxin has an unusual capability in causing rapid and strong phosphorylation at S729 and triggering B cells to express spliced XBP1. To assess the function of S729 in IRE1, we generated S729A knock-in mice and found S729 is critically important for lipopolysaccharide-stimulated plasmablasts to respond to additional ER stress and for antibody production in response to immunization. We further crossed mice carrying an S729A mutation or ΔIRE1 (missing the kinase domain) with B cell–specific XBP1-deficient mice to trigger RIDD and discovered a critical role for S729 in regulating RIDD in B cells.

## Abstract (precis)

Phosphorylation of IRE1 at S729 enhances splicing of XBP1 messenger RNA and regulates RIDD. lipopolysaccharide-stimulated plasmablasts from S729A knock-in mice fail to boost spliced XBP1 in response to ER stress. Such mice exhibit plasma cells with decreased numbers and altered functions after immunization.

## Introduction

The ER is responsible for the folding and assembly of ∼30% of proteins encoded by our genome. Many secretory and membrane-bound proteins are important cytokines and surface receptors. The ER harbors complex, yet elegant, mechanisms to control protein folding and assembly and to dispose of terminally misfolded proteins. To respond to ER stress, the ER is equipped with transmembrane sensors IRE1, PERK, and ATF6, representing the three major arms of the unfolded protein response (UPR), which help cells relieve the stress and restore homeostasis (Ron and Walter, 2007; Walter and Ron, 2011). In the case of persistent and irreversible stress, the ER can also dictate cell death. Aberrant regulation of the UPR is implicated in many diseases (Lin et al., 2008; Hetz et al., 2013; Hetz and Mollereau, 2014; Bettigole and Glimcher, 2015; Chevet et al., 2015; Grootjans et al., 2016).

The ER stress sensor, IRE1, is critical for B cells. Normal B cell development in the bone marrow requires IRE1 (Zhang et al., 2005). Upon encountering its cognate antigen, a B cell differentiates into a plasma cell, which can produce large quantities of high-affinity antibodies against the antigen. IRE1 is indispensable in this process because its cytoplasmic kinase/RNase domain, upon stimulation for differentiation, can assemble into a functional RNase that specifically splices 26 nucleotides from mammalian XBP1 mRNA (Shen et al., 2001; Yoshida et al., 2001; Calfon et al., 2002; Korennykh et al., 2009). The spliced XBP1 (XBP1s) mRNA encodes a functional 54-kD transcription factor, XBP1s, as a result of a frame shift in translation (Calfon et al., 2002). XBP1s up-regulates the synthesis of lipids and chaperones, contributing to the ER expansion and increased Ig production in plasma cells (Lee et al., 2003; Sriburi et al., 2004; McGehee et al., 2009). In response to Toll-like receptor (TLR) ligands such as lipopolysaccharide (LPS; a TLR4 ligand) or cytosine-phosphate-guanine (CpG) DNA (a TLR9 ligand), B cells activate the IRE1–XBP1 pathway and produce large quantities of secretory IgM (sIgM; Reimold et al., 2001; Iwakoshi et al., 2003; Hu et al., 2009a). Data showing vastly decreased sIgM in stimulated IRE1- (Zhang et al., 2005) and XBP1-deficient B cells (Reimold et al., 2001; Iwakoshi et al., 2003; Hu et al., 2009a) support the role of the IRE1–XBP1 pathway in antibody production.

Other than splicing XBP1 mRNA, the RNase of IRE1 can rapidly cleave a subset of mRNAs and so halts the production of proteins that challenge the ER. This mechanism is known as regulated IRE1-dependent decay (RIDD; Hollien and Weissman, 2006). We showed previously that genetic deletion of the *XBP1* gene leads to elevated protein levels of IRE1 in B cells (Hu et al., 2009a). Recently, the mRNA of secretory Ig μ (μS) heavy chain was shown to be an RIDD substrate, and increased levels of IRE1 in XBP1-deficient B cells contribute to decreased levels of sIgM by cleaving μS mRNA (Benhamron et al., 2014). Ablation of the RNase activity of IRE1 in XBP1-deficent B cells inhibits the RIDD of μS mRNA (Benhamron et al., 2014). RIDD is clearly important in B cells because it protects XBP1-deficient B cells from accumulating unfolded proteins in the ER by degrading μS mRNA, which encodes one of the most abundant ER proteins in B cells. Enhanced RIDD in response to XBP1 deficiency is also critical in regulating proinsulin processing and insulin secretion in pancreatic β-cells (Lee et al., 2011), protecting hepatocytes from acetaminophen-induced hepatotoxicity (Hur et al., 2012) and suppressing lipogenesis and lipoprotein metabolism (So et al., 2012). In response to prolonged ER stress, RIDD is responsible for the decay of specific microRNAs that repress translation of the caspase-2 mRNA, causing drastically elevated levels of caspase-2 (Upton et al., 2012).

We identified that IRE1 is phosphorylated at S729 in XBP1-deficient mouse B cells. By generating and using a specific anti–phospho-S729 antibody, we confirmed that S729 is indeed phosphorylated in XBP1-deficient B cells and discovered that phosphorylation of S729 only occurs under certain ER stress conditions. Next, we generated a knock-in mouse model, S729A, and showed that, although B cells from mice carrying the S729A mutation can respond to LPS-stimulated B cell differentiation by producing XBP1s, they fail to respond to additional pharmacologic or bacterial toxin insults. To investigate the role of S729 and the kinase domain of IRE1 in regulating RIDD, we crossed S729A and IRE1^f/f^ mice with B cell-specific XBP1 knockout (KO; XBP1^KO^; CD19Cre/XBP1^f/f^) mice to trigger RIDD. Our results showed that S729 phosphorylation of IRE1 is critical for enhancing the splicing of XBP1 mRNA and engaging RIDD in cultured B cells and plasma cells in immunized mice.

## Results

### IRE1 responded to XBP1 deficiency by undergoing phosphorylation at S729

**Figure 1.**
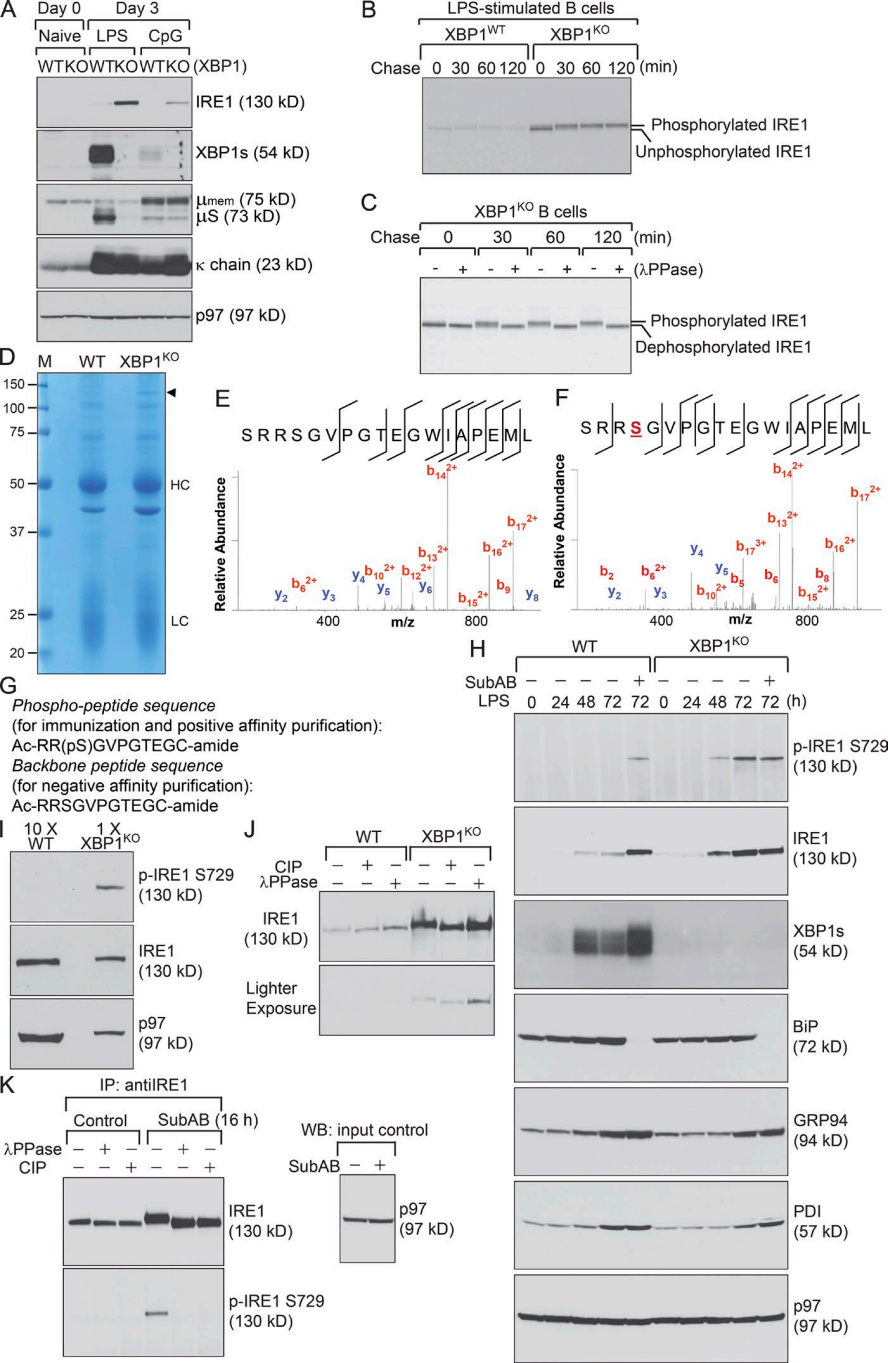
**IRE1 is phosphorylated at S729 in XBP1-deficient B cells. (A)** Naive XBP1^WT^ and XBP1^KO^ B cells were stimulated with LPS (20 µg/ml) or CpG-1826 (0.5 µM) for 3 d. Lysates were immunoblotted for indicated proteins. **(B)** 3-d LPS-stimulated XBP1^WT^ and XBP1^KO^ B cells were starved in cysteine- and methionine-free media for 1 h, radiolabeled for 15 min, and chased for a course of 2 h. Lysates were immunoprecipitated (IP) for IRE1, which was then analyzed by SDS-PAGE and autoradiography. **(C)** 3-d LPS-stimulated XBP1^KO^ B cells were starved for 50 min, radiolabeled for 10 min, and chased. Lysates were immunoprecipitated for IRE1, which was boiled in 1% SDS containing 5 mM DTT, further diluted to contain 0.05% SDS and 0.5% NP-40, and reimmunoprecipitated using the same anti–IRE1 antibody. Bead-bound IRE1 was further treated with λPPase for 3 h and analyzed by SDS-PAGE and autoradiography. **(D)** LPS-stimulated XBP1^WT^ and XBP1^KO^ B cell lysates were immunoprecipitated for IRE1, analyzed by SDS-PAGE, and stained with Coomassie brilliant blue G-250. The closed triangle marks IRE1, confirmed by LC-MS/MS sequencing. HC, IgG heavy chains; LC, IgG light chains; M, molecular weight. **(E)** The unmodified peptide, aa 726–743, was detected as a triply protonated molecule at m/z 648.3312 (−0.71 ppm error) and assigned with a MASCOT score of 50. **(F)** The corresponding phosphopeptide was detected at 674.9878 (−1.3 ppm error) with a MASCOT score of 35. The ions in the tandem spectra are labeled and marked in the sequence. S indicates the phosphorylation. **(G)** Amino acid sequences of the phosphopeptide and backbone peptide of IRE1 for generation and purification of the anti–phospho-S729 antibody. **(H)** XBP1^WT^ and XBP1^KO^ B cells were stimulated with LPS for 72 h. Some LPS-stimulated B cells were exposed to SubAB in the last 24 h of the time course. Lysates were immunoblotted for the indicated proteins or immunoprecipitated with an anti–mouse IRE1 antibody before immunoblotting with the anti–phospho-S729 antibody. **(I)** XBP1^WT^ and XBP1^KO^ B cells were stimulated with LPS for 72 h. 10 times more XBP1^WT^ B cell lysates were used for immunoprecipitation for IRE1, which was then immunoblotted with the anti–phospho-S729 antibody. IRE1 and p97 immunoblots are input controls. **(J)** 3-d LPS-stimulated XBP1^WT^ and XBP1^KO^ B cells were lysed and immunoprecipitated for IRE1. Bead-bound IRE1 was treated with CIP or λPPase for 3 h and immunoblotted for IRE1. **(K)** 5TGM1 myeloma cells were exposed to SubAB for 16 h. Lysates were immunoprecipitated for IRE1. Bead-bound IRE1 was treated with λPPase or CIP for 3 h and immunoblotted for the indicated proteins. The p97 immunoblot is an input control. Data in this figure are representative of at least three independent experiments. WB, Western blot.

To investigate the role of IRE1 in XBP1-deficient B cells, we stimulated naive B cells purified from the spleens of XBP1^WT^ and XBP1^KO^ mice with LPS and CpG-1826. Both LPS- and CpG-1826–stimulated B cells produced large quantities of Ig κ light chains (Fig. 1 A). LPS-stimulated B cells produced significantly more sIgM than did CpG-1826–stimulated B cells. CpG-1826–stimulated B cells produced more membrane-bound IgM. Although the lack of XBP1 in B cells did not affect the production of membrane-bound IgM or κ light chains, it led to significantly suppressed expression of sIgM, which was attributed to increased expression levels of IRE1 and RIDD (Fig. 1 A; Benhamron et al., 2014). The expression level of IRE1 was up-regulated after stimulation with LPS or CpG-1826 (Fig. 1 A; Hu et al., 2009a). To compare the synthesis of IRE1 in LPS-stimulated XBP1-proficient and -deficient B cells, we pulse-labeled the cells using [^35^S]methionine and [^35^S]cysteine for 15 min, performed a 2-h chase experiment in the presence of cold methionine and cysteine, and immunoprecipitated IRE1 using an anti–mouse IRE1 antibody generated in house. In contrast with XBP1-proficient B cells, XBP1-deficient B cells produced more IRE1, which shifted to a higher molecular weight after a 30-min chase and thereafter (Fig. 1 B). We demonstrated that the molecular weight shift of IRE1 was a result of phosphorylation by performing a pulse-chase experiment coupled with digestion of the immunoprecipitated IRE1 using λ protein phosphatase (λPPase; Fig. 1 C).

To identify the phosphorylated residues of IRE1, we immunoprecipitated IRE1 from LPS-stimulated, XBP1-deficient and -proficient B cells using the anti–IRE1 antibody and analyzed the immunoprecipitates by SDS-PAGE. IRE1 and other proteins were visualized by staining with Coomassie brilliant blue (G-250; Fig. 1 D). The band corresponding to the molecular weight of IRE1 was excised from the gel, in-gel digested, and subjected to peptide sequencing by liquid chromatography–tandem mass spectrometry (LC-MS/MS). Single-peptide matches for protein identifications and all phosphorylated peptides were further verified by manual inspection of the tandem mass spectra (and relatively quantified using extracted ion chromatograms [XICs]). Most peptides detected in our LC-MS/MS analysis belonged to IRE1. By using various enzymes (trypsin, chymotrypsin, or both combined) for proteolytic digestion in different experiments, we recovered peptides that covered >95% of the mouse IRE1 cytoplasmic domain sequence for a relatively thorough phosphorylation analysis. By XIC analysis, we detected clear phosphorylation of S729 in IRE1 in response to XBP1 deficiency (Fig. 1, E and F), with no evidence suggesting that S724 or S726 was phosphorylated in multiple LC-MS/MS analyses, and by using a commercial antibody against phospho-S724.

To investigate the role of S729, we immunized rabbits with a synthetic IRE1 peptide containing phosphorylated S729 residue (Fig. 1 G) and affinity-purified anti–phospho-S729 antibodies using exactly the same peptide. The antibodies were further purified using a synthetic backbone peptide (Fig. 1 G) to deplete those that recognize nonphosphorylated IRE1. To characterize our anti–phospho-S729 antibody, we treated naive XBP1-proficient and -deficient B cells with LPS for a course of 72 h, immunoprecipitated IRE1 using an anti–mouse IRE1 antibody, and showed that this antibody recognizes the 130-kD phospho-IRE1 protein produced by 48-h and 72-h LPS-stimulated XBP1-deficient B cells (Fig. 1 H). To examine whether S729 phosphorylation occurred in LPS-stimulated XBP1-proficient B cells, we immunoprecipitated IRE1 from 10 times more XBP1-proficient B cell lysates so that the backbone IRE1 became comparable with that in XBP1-deficient B cell lysates. We did not detect S729 phosphorylation of IRE1 in XBP1-proficient cell lysates by immunoblots (Fig. 1 I). We also treated immunoprecipitated IRE1 with calf intestinal phosphatase (CIP) or λPPase and showed that IRE1 did not undergo phosphorylation in LPS-stimulated XBP1-proficient B cells (Fig. 1 J), consistent with pulse-chase data (Fig. 1 B). We have shown that subtilase cytotoxin (SubAB) produced by Shiga-toxigenic *Escherichia coli* specifically cleaved BiP (Paton et al., 2006) and led to phosphorylation of IRE1 (Hu et al., 2009b); however, the phosphorylated residue was not known. We treated 48-h LPS-stimulated XBP1-proficient and -deficient B cells with SubAB for 24 h and showed that our anti–phospho-S729 antibody recognized phosphorylated IRE1 in SubAB-treated B cells (Fig. 1 H). To confirm the phosphospecificity of our anti–phospho-S729 antibody, we treated 5TGM1 mouse multiple myeloma cells with SubAB immunoprecipitated IRE1 using an anti–IRE1 antibody and incubated immunoprecipitated IRE1 with λPPase or CIP before immunoblotting using the anti–phospho-S729 antibody. This antibody recognized phosphorylated IRE1 in SubAB-treated 5TGM1 cells and did not react with that preincubated with λPPase or CIP (Fig. 1 K).

### SubAB was potent in inducing S729 phosphorylation of IRE1, which could be rapidly suppressed by KIRA6

**Figure 2.**
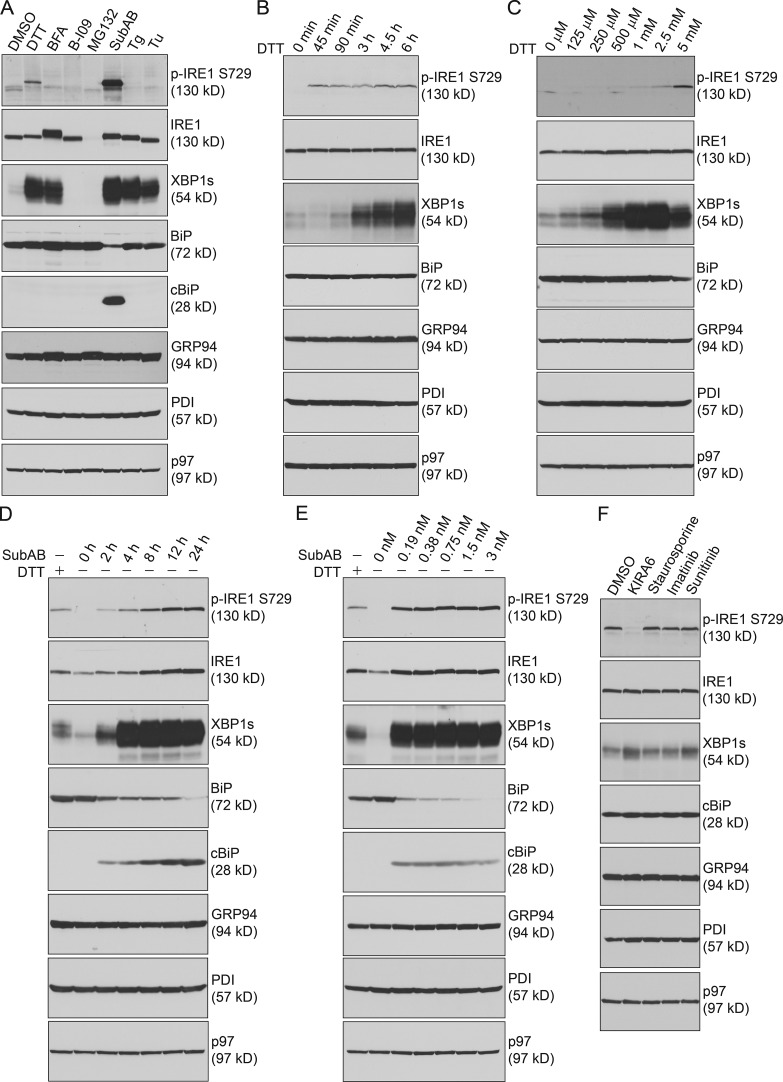
**DTT and SubAB induce IRE1 to undergo strong and rapid phosphorylation at S729. (A)** 5TGM1 cells were treated with 5 mM DTT for 3 h or with 3.5 µM BFA, 20 µM B-I09, 50 µM MG132, 1 nM SubAB, 2.5 µM Tg, or 5 µg/ml Tu for 12 h. Lysates were immunoblotted for the indicated proteins. **(B)** 5TGM1 cells were treated with 5 mM DTT for the indicated times and immunoblotted. **(C)** 5TGM1 cells were treated with DTT at the indicated concentrations for 3 h and immunoblotted. **(D)** 5TGM1 cells were treated with 5 mM DTT for 3 h or with 1.5 nM SubAB for 24 h and immunoblotted. **(E)** 5TGM1 cells were treated with 5 mM DTT for 3 h or with SubAB for 12 h using increasing concentrations and immunoblotted. **(F)** 5TGM1 cells were exposed to 1 nM SubAB for 12 h and were subsequently treated with KIRA6, staurosporine, imatinib, or sunitinib at 5 µM for an additional 2 h and then immunoblotted. Data in this figure are representative of three independent experiments.

To investigate the role of S729 phosphorylation, we treated 5TGM1 cells with DTT, brefeldin A (BFA), B-I09 (an IRE1 RNase inhibitor; Tang et al., 2014), MG132, SubAB, thapsigargin (Tg), or tunicamycin (Tu; Fig. 2 A). We performed time-course experiments using 5 mM DTT, based on our result showing that 5 mM DTT induced the highest phosphorylation at S729 of IRE1 in dose-dependent experiments, and observed that S729 phosphorylation occurred at the 45-min time point, whereas significantly increased XBP1s occurred at the 3-h time point (Fig. 2, B and C). We then performed time-course and dose-dependent experiments for SubAB (Fig. 2, D and E), Tg (Fig. S1, A and B), Tu (Fig. S1, C and D), BFA (Fig. S1, E and F), and MG132 (Fig. S1, G and H) and compared results with those treated with 5 mM DTT for 3 h in all experiments. Compared with DTT, 1.5 nM SubAB cleaved BiP and induced increased synthesis and phosphorylation of IRE1 accompanied by the expression of XBP1s in the course of 24-h treatment (Fig. 2 D). Phosphorylation of S729 was also detected in experiments using lower concentrations of SubAB (Fig. 2 E). Tg induced S729 phosphorylation to a much lesser degree than DTT in both time-course and dose-dependent experiments (Fig. S1, A and B). In repeated time-course and dose-dependent experiments, we did not detect S729 phosphorylation in cells treated with Tu (Fig. S1, C and D), BFA (Fig. S1, E and F), or MG132 (Fig. S1, G and H). 5TGM1 cells treated with MG132 expressed reduced levels of IRE1 after 4 h (Fig. S1 G) because of rapid apoptosis but not lysosomal degradation (Fig. S1 I); however, those treated with BFA or Tu at least maintained, if not increased, the expression levels of IRE1 (Fig. S1, C–F). Untreated 5TGM1 cells as well as those treated with Tu, BFA, or MG132 all expressed XBP1s despite the lack of S729 phosphorylation (Fig. S1, C–H), suggesting that S729 phosphorylation of IRE1 was not required for initial activation of XBP1s. We immunoprecipitated IRE1 from untreated as well as Tu- and BFA-treated 5TGM1 cells, dephosphorylated IRE1 using CIP, and showed that IRE1 in untreated or Tu- or BFA-treated 5TGM1 cells was phosphorylated at other sites (Fig. S1, J and K).

KIRA6, an IRE1 kinase inhibitor, was shown to inhibit IRE1 from splicing XBP1 mRNA and cleaving insulin mRNA (Ghosh et al., 2014). We tested whether KIRA6 could affect S729 phosphorylation of IRE1 by treating 5TGM1 cells with SubAB in the presence of increasing concentrations of KIRA6 for 12 h (Fig. S2 A). KIRA6 suppressed SubAB-induced S729 phosphorylation of IRE1 in a dose-dependent manner, with little impact on the levels of XBP1s. In addition, we treated 5TGM1 cells with SubAB for 12 h and then exposed such cells to 5 µM KIRA6 for a course of 8 h (Fig. S2 B). KIRA6 rapidly suppressed S729 phosphorylation without affecting the levels of XBP1s. KIRA6 also did not suppress phosphorylation of IRE1 at other sites (Fig. S2 C). Compared with staurosporine (a broad-spectrum kinase inhibitor that binds to IRE1; Concha et al., 2015), imatinib (a tyrosine kinase inhibitor that activates IRE1 in cardiomyocytes; Kerkelä et al., 2006), or sunitinib (a receptor tyrosine kinase inhibitor that binds to IRE1; Korennykh et al., 2009; Ali et al., 2011), KIRA6 was effective in suppressing S729 phosphorylation of IRE1 (Figs. 2 F and S2 D).

### SubAB but not other AB_5_ toxins induced S729 phosphorylation of IRE1

**Figure 3.**
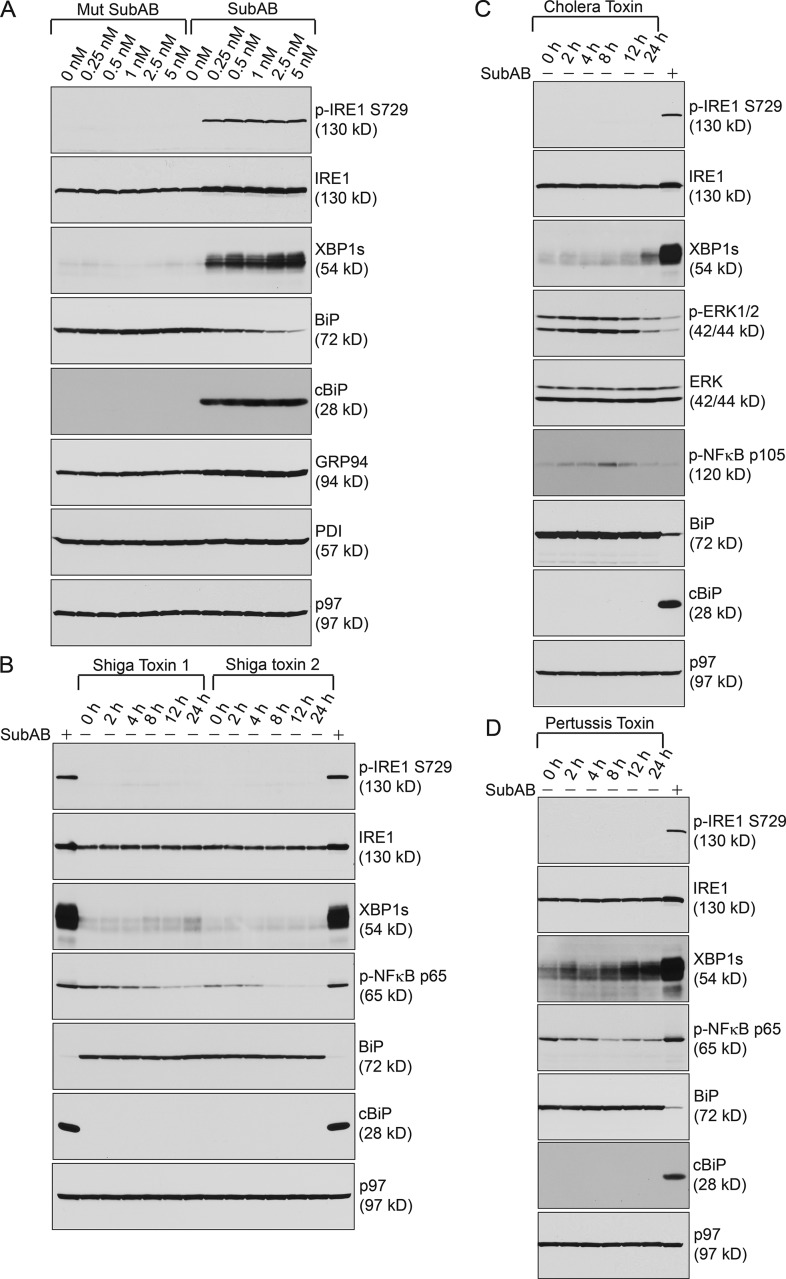
**SubAB, but not other AB_5_ toxins, cleaves BiP and induces S729 phosphorylation of IRE1. (A)** 5TGM1 cells were treated with mutant or native SubAB for 24 h using increasing concentrations and immunoblotted. **(B)** 5TGM1 cells were treated with 1 nM SubAB, 1 nM Shiga toxin 1, or 1 nM Shiga toxin 2 for 24 h and immunoblotted. Phospho–NF-κB p65 served as a control for the activity of the Shiga toxins. **(C)** 5TGM1 cells were treated with 1 nM SubAB for 24 h or with 1 nM cholera toxin for 24 h and immunoblotted. Phospho-ERK1/2 and phospho–NF-κB p105 served as controls for the activity of the cholera toxin. **(D)** 5TGM1 cells were treated with 1 nM SubAB or 1 nM pertussis toxin for 24 h and immunoblotted. Phospho–NF-κB p65 served as a control for pertussis toxin activity. Data in this figure are representative of three independent experiments.

Cytotoxicity of SubAB is a result of BiP cleavage at a dileucine motif (Leu417 and Leu418 in mouse BiP); overexpression of BiP in which the SubAB cleavage site is eliminated can protect cells from SubAB-induced cytotoxicity (Paton et al., 2006). To demonstrate that SubAB induced S729 phosphorylation through cleaving BiP, we treated 5TGM1 cells with a mutant SubA_A272_B carrying a single serine-to-alanine substitution at the critical Ser272 in the catalytic triad (Asp-His-Ser) of its A subunit and showed that only native SubAB cleaved BiP and caused S729 phosphorylation of IRE1 (Fig. 3 A). Other than SubAB, there are three types of AB_5_ toxins: Shiga toxins 1 and 2 from *Shigella dysenteriae* and *E. coli*, cholera toxin from *Vibrio cholera*, and pertussis toxin from *Bordetella pertussis*. Similar to SubAB, all three AB_5_ toxins enter the ER through retrograde transport. Although SubAB cleaves BiP in the ER, the other AB_5_ toxins need to be retrotranslocated from the ER into the cytoplasm to exert their toxic effects. We treated 5TGM1 cells with Shiga toxins 1 and 2 (Figs. 3 B and S2 E), cholera toxin (Figs. 3 C and S2 F), or pertussis toxin (Figs. 3 D and S2 G) and investigated the capabilities of these toxins in inducing S729 phosphorylation of IRE1. Even when we treated 5TGM1 cells with 10 times more concentrated Shiga toxins, cholera toxin or pertussis toxin than 1 nM SubAB, we did not detect S729 phosphorylation of IRE1 (Fig. S2, E–G). However, we observed slightly increased expression of XBP1s in Shiga toxin–, cholera toxin–, and pertussis toxin–treated cells (Fig. 3, B–D).

### Generation of the S729A knock-in mouse model

**Figure 4.**
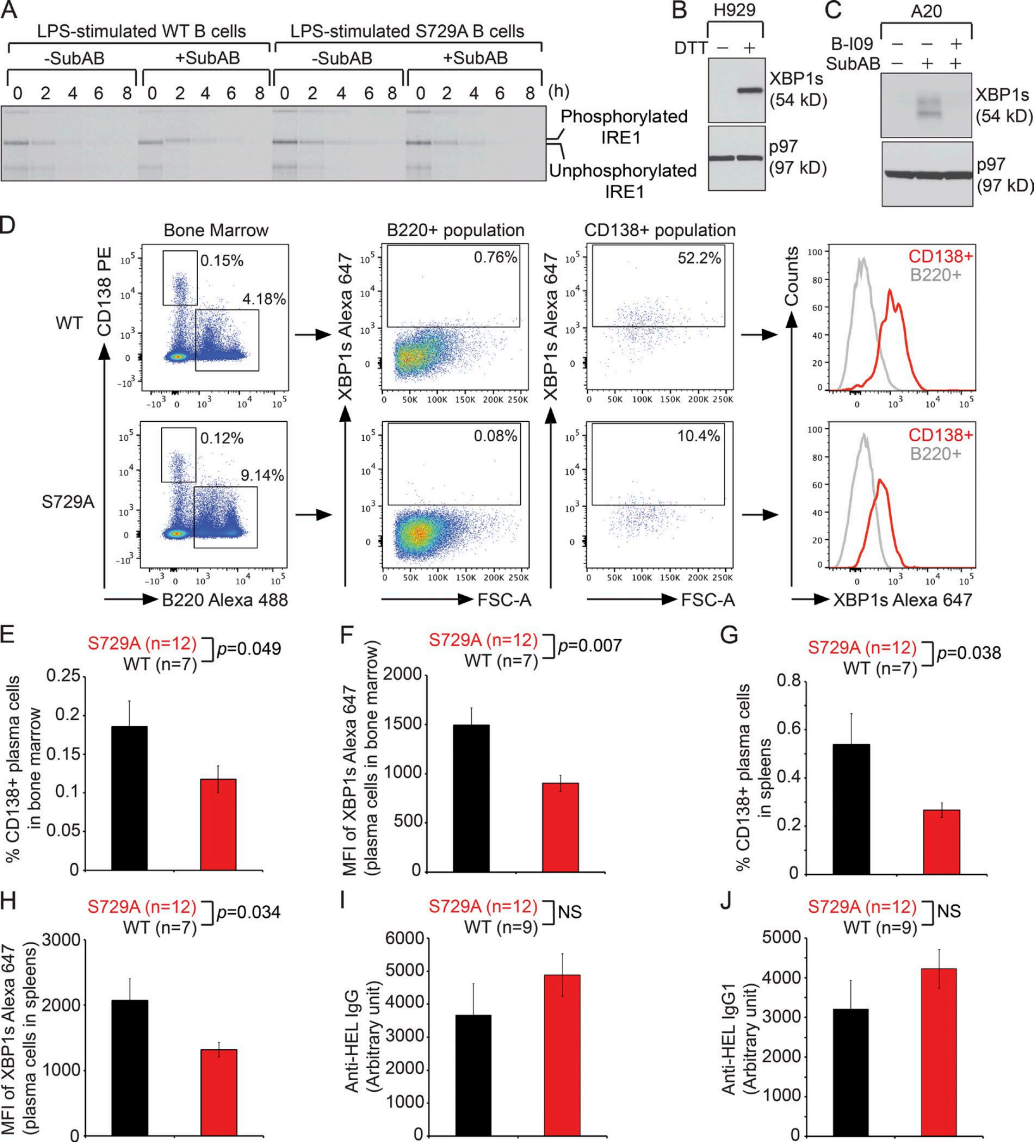
**HEL-immunized S729A mice generate significantly less CD138^+^ plasma cells in the spleens and bone marrow, and those plasma cells express less XBP1s. (A)** 3-d LPS-stimulated WT and S729A B cells were radiolabeled for 4 h and chased in the absence or presence of 1 nM SubAB for a course of 8 h. Lysates were immunoprecipitated for IRE1, which was then analyzed by SDS-PAGE and autoradiography. **(B)** Human H929 cells were treated with 5 mM DTT for 3 h and immunoblotted. **(C)** Mouse A20 cells were pretreated with 20 µM B-I09 for 12 h, were subsequently exposed to 1.5 nM SubAB for 12 h, and were immunoblotted. **(D)** WT and S729A mice were immunized with HEL plus CFA and boosted three times with HEL plus IFA. Bone marrow cells isolated from immunized mice were stained for CD138, B220, and XBP1s. Gated B220^+^ and CD138^+^ populations were analyzed for expression of XBP1s. FSC-A, forward scatter area. **(E)** Percentages of CD138^+^ plasma cells in the bone marrow of HEL-immunized WT and S729A mice. **(F)** MFI of XBP1s in CD138^+^ plasma cells in the bone marrow of HEL-immunized WT and S729A mice. **(G)** Percentages of CD138^+^ plasma cells in spleens of HEL-immunized WT and S729A mice. **(H)** MFI of XBP1s in CD138^+^ plasma cells in spleens of HEL-immunized WT and S729A mice. **(I and J)** Serum levels of anti–HEL IgG (I) and IgG1 (J) in immunized WT and S729A mice were determined by ELISA. Data are shown as means ± SEM.

To investigate the physiological role of the S729 residue of IRE1, we generated a novel knock-in mouse model, S729A (Fig. S3 A). We confirmed the long- and short-arm integration of the targeting vector by Southern blots (Fig. S3 A) and the presence of the desired mutation in the *IRE1* gene by DNA sequencing. Purified B cells from WT and S729A mice were cultured in the presence of LPS for 3 d, radiolabeled for 4 h, and chased for a course of 8 h in the absence or presence of SubAB, and the immunoprecipitation results confirmed that LPS could not trigger IRE1 to undergo phosphorylation at S729 in WT B cells, but SubAB could (Fig. 4 A).

### The percentages of B and T cells changed slightly in S729A knock-in mice

To examine B cell development in the bone marrow of S729A mice, we analyzed gated B220^+^ B cell progenitors for CD43^+^/CD19^low^ pro–B cells and CD43^+^/CD19^high^ pre–B cells (Fig. S3 B, middle) and detected little change in the percentages of B cell progenitors, pro–, or pre–B cells when comparing unimmunized S729A with WT mice (Fig. S3, B–E). We also gated the CD43^−^/CD19^+^ population and analyzed them for IgM^+^/IgD^−^ immature B cells and IgM^+^/IgD^+^ mature B cells in the bone marrow (Fig. S3 B, right), and we found that S729A mice exhibit similar percentages of immature and mature B cells when compared with WT mice (Fig. S3, F and G). WT and S729A mice were also immunized with hen egg lysozyme (HEL), emulsified in complete Freund’s adjuvant (CFA), and boosted three times with HEL plus incomplete Freund’s adjuvant (IFA). We observed increased numbers of pro– and mature B cells in the bone marrow of S729A mice (Fig. S3, D and G).

In the spleens of unimmunized and HEL-immunized mice, we analyzed the gated CD19^+^ B cell population and detected no difference of this population between WT and S729A mice (Fig. S4, A and B). We next gated CD3^+^ populations to analyze for CD4^+^ and CD8^+^ T cells. Normal CD4/CD8 T cell ratio and T cell percentages were found in unimmunized WT and S729A mice (Fig. S4, A, C, and D); however, increased percentages of CD4^+^ and CD8^+^ T cells were observed in immunized S729A mice (Fig. S4, C and D). We also analyzed the gated CD19^+^/B220^+^/GL7^−^/AA4.1^−^ B cell population for CD1d^+^/CD23^−^ marginal zone B cells and CD1d^−^/CD23^+^ follicular B cells in spleens and found no difference between unimmunized and immunized WT and S729A mice (Fig. S4, E–G).

In peripheral lymph nodes of unimmunized and HEL-immunized mice, we analyzed the gated CD19^+^ B cell population and found decreased percentages of this population in immunized S729A mice (Fig. S4, H and I). We also analyzed the gated CD3^+^ populations for CD4^+^ and CD8^+^ T cells and detected increased CD4 and CD8 T cell percentages in the lymph nodes of immunized S729A mice (Fig. S4, H, J, and K).

### Immunized S729A mice generated less CD138^+^ plasma cells, which expressed lower levels of XBP1s

To compare the expression levels of XBP1s in plasma cells from immunized WT and S729A mice, we characterized a recently developed anti-XBP1s mouse monoclonal antibody. This antibody recognized human XBP1s expressed by H929 myeloma cells stimulated with DTT and mouse XBP1s produced by A20 B cell lymphoma upon SubAB stimulation (Fig. 4, B and C). As a negative control for this antibody, B-I09 abolished SubAB-induced expression of XBP1s in A20 cells (Fig. 4 C). We stained bone marrow cells from HEL-immunized WT and S729A mice for CD138, B220, and XBP1s and found that CD138^+^ plasma cells indeed produced higher levels of XBP1s than B220^+^ B cell progenitors (Fig. 4 D). When comparing immunized WT and S729A mice, we found decreased percentages of CD138^+^ plasma cells in the bone marrow of S729A mice (Fig. 4, D and E) and significantly lower mean fluorescence intensity (MFI) of XBP1s expressed by CD138^+^ plasma cells in immunized S729A mice (Fig. 4 F). We also found decreased percentages of CD138^+^ plasma cells in spleens of immunized S729A mice (Fig. 4 G), and these plasma cells similarly expressed lower levels of XBP1s (Fig. 4 H). Despite the reduced percentages of plasma cells in HEL-immunized S729A mice, serum levels of anti–HEL IgG or IgG1 remained unabated (Fig. 4, I and J).

### S729A B cells responded to LPS by producing XBP1s but could not respond to additional ER insults by up-regulating the levels of XBP1s

**Figure 5.**
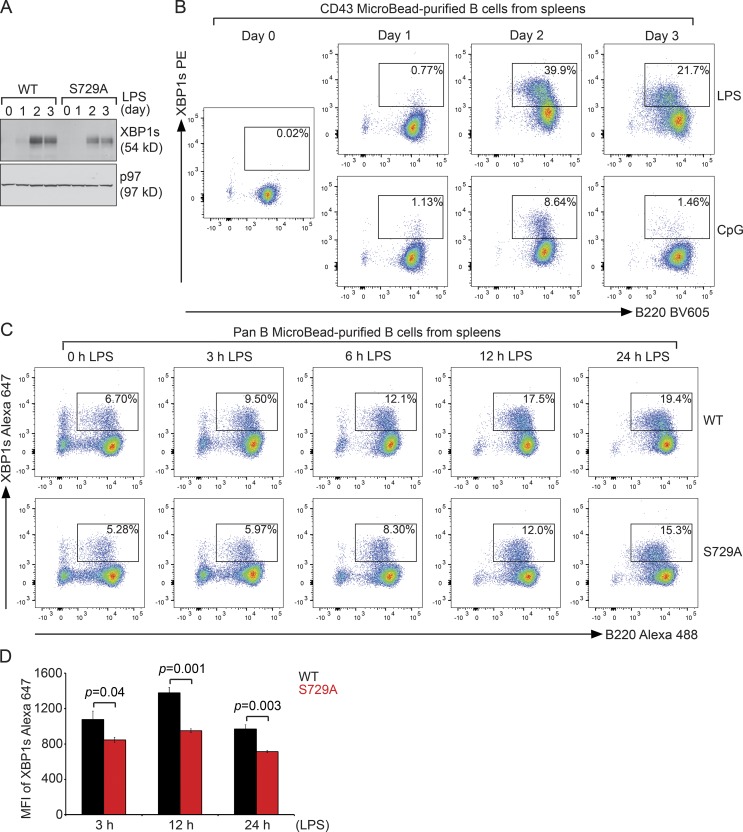
**S729A B cells respond to LPS or CpG-1826 stimulation by producing XBP1s. (A)** Naive WT and S729A B cells were stimulated with LPS (20 µg/ml) for a course of 3 d and immunoblotted. **(B)** Naive WT B cells were stimulated with LPS or CpG-1826 (0.5 µM) for a course of 3 d, stained for XBP1s and B220, and analyzed for gated B220^+^/XBP1s^+^ populations. **(C)** B cells purified from WT and S729A mice were stimulated with LPS for 1 d and continued to be stimulated with LPS for an additional 3, 6, 12, and 24 h (the end of day 2). At each time point, B cells were stained for XBP1s and B220 and were analyzed for gated B220^+^/XBP1s^+^ populations. Flow cytofluorometric data are representative of three independent experiments. **(D)** Naive B cells purified from WT (*n* = 3) and S729A (*n* = 3) mice were stimulated with LPS for 1 d and continued to be stimulated with LPS for an additional 3, 12, and 24 h. At each time point, B cells were stained with XBP1s–Alexa Fluor 647 and B220–Alexa Fluor 488. The gated B220^+^ populations were analyzed for expression of XBP1s shown as MFI (means ± SD). The MFI data are representative of three independent experiments.

To further analyze B cell differentiation, we purified naive B cells from S729A mice, stimulated them with LPS, and found that S729A B cells can express XBP1s, albeit at decreased levels (Fig. 5 A). By analyzing LPS- or CpG-1826–stimulated B cells stained with the anti-XBP1s antibody, we realized for the first time that not all naive B cells can respond to LPS or CpG-1826 by producing high levels of XBP1s (Fig. 5 B). Compared with CpG-1826, LPS induced significantly more B cells to produce XBP1s during 3 d of stimulation (Fig. 5 B), consistent with our immunoblot data (Fig. 1 A). Next, we stimulated B cells purified from S729A mice with LPS for 24 h and monitored the percentages of cells that expressed XBP1s as well as the MFI of XBP1s by flow cytofluorometry for an additional 24 h. Compared with WT B cells, S729A B cells expressed slightly lower levels of XBP1s (Fig. 5, C and D), consistent with our immunoblot data (Fig. 5 A).

**Figure 6.**
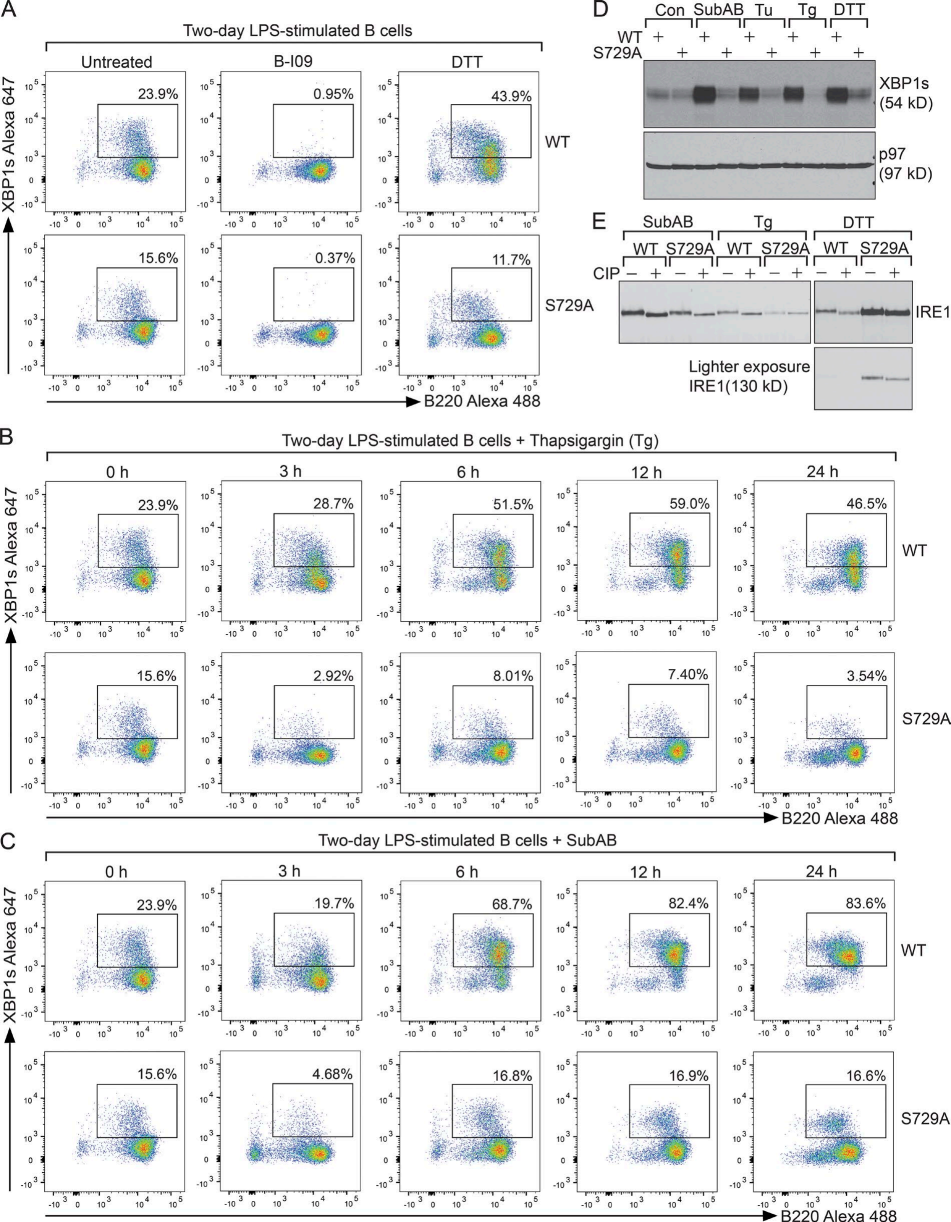
**LPS-stimulated S729A B cells fail to respond to secondary ER stress by producing additional XBP1s. (A)** 2-d LPS-stimulated WT and S729A B cells were exposed to 20 µM B-I09 or 5 mM DTT for 3 h, stained for XBP1s and B220, and gated for B220^+^/XBP1s^+^ populations. **(B)** 2-d LPS-stimulated WT and S729A B cells were exposed to 2.5 µM Tg for the indicated times, stained for XBP1s and B220, and gated for B220^+^/XBP1s^+^ populations. **(C)** 2-d LPS-stimulated WT and S729A B cells were exposed to 1.5 nM SubAB for the indicated times, stained for XBP1s and B220, and gated for B220^+^/XBP1s^+^ populations. **(D)** 2-d LPS-stimulated WT and S729A B cells were exposed to 1 nM SubAB, 5 µg/ml Tu, or 2.5 µM Tg for 12 h or were exposed to 5 mM DTT for 3 h and immunoblotted. **(E)** 2-d LPS-stimulated WT and S729A B cells were exposed to 1 nM SubAB or 2.5 µM Tg for 12 h or were exposed to 5 mM DTT for 3 h, lysed, and immunoprecipitated for IRE1. Bead-bound IRE1 was treated with CIP for 3 h and immunoblotted for IRE1. Data in this figure are representative of three independent experiments.

To explore how S729A B cells responded to additional ER insults, we exposed 2-d LPS-stimulated WT and S729A B cells to DTT for 3 h or to Tg or SubAB for 3, 6, 12, and 24 h, and monitored the percentages of XBP1s-expressing B cells and the MFI of XBP1s by flow cytofluorometry (Fig. 6, A–C; and Fig. S5, A and B). As a negative control, LPS-stimulated B cells were treated with B-I09 to shut down the expression of XBP1s (Fig. 6 A). Compared with DTT or Tg (Fig. 6, A and B; and Fig. S5 A), SubAB triggered most of the LPS-stimulated WT B cells to further express XBP1s, resulting in much higher MFI (Figs. 6 C and S5 B). However, all these ER stress inducers failed to trigger LPS-stimulated S729A B cells to further express XBP1s (Fig. 6, A–C; and Fig. S5, A and B), and we confirmed such results by immunoblots (Fig. 6 D). In addition, IRE1 immunoprecipitated from SubAB- and DTT- but not Tg-treated S729A B cells could be further dephosphorylated with CIP (Fig. 6 E), suggesting that SubAB and DTT could also induce phosphorylation of IRE1 at sites different from S729. Nevertheless, in the face of S729A mutation, the enhanced expression of XBP1 in response to SubAB or DTT was completely blocked (Fig. 6, A, C, and D).

### Phosphorylation at S729 of IRE1 was responsible for RIDD

**Figure 7.**
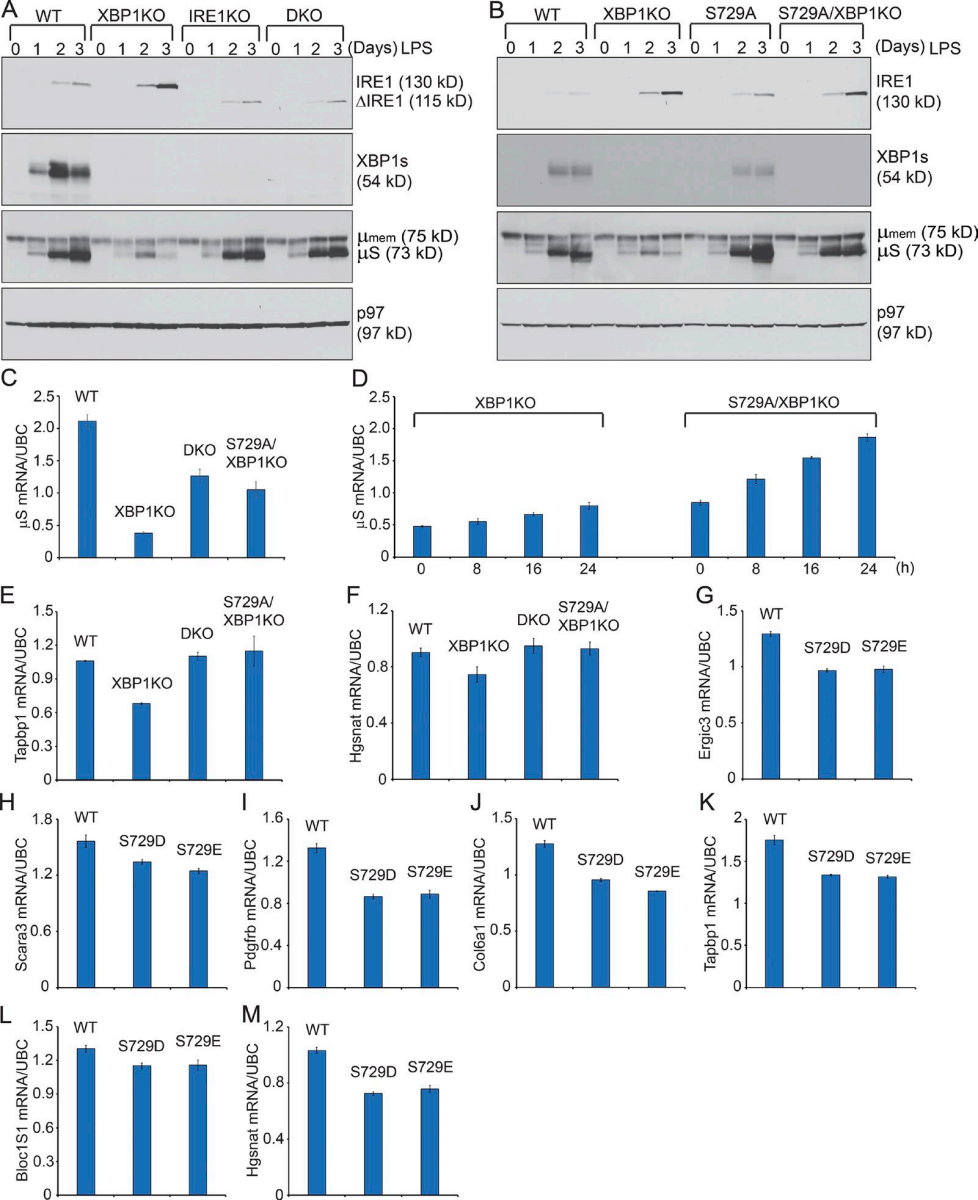
**Phosphorylation at S729 of IRE1 is critical for RIDD. (A)** WT, XBP1^KO^, IRE1^KO^, and IRE1^KO^/XBP1^KO^ (double KO; DKO) naive B cells were stimulated with 20 µg/ml LPS for a course of 3 d and immunoblotted. **(B)** WT, XBP1^KO^, S729A, and S729A/XBP1^KO^ naive B cells were stimulated with LPS for a course of 3 d and immunoblotted. Immunoblot data shown in this figure are representative of three independent experiments. **(C)** Naive B cells from WT, XBP1^KO^, DKO, and S729A/XBP1^KO^ mice were stimulated with LPS for 3 d and lysed for purification of total RNA and synthesis of cDNA. The levels of μS mRNA were measured by quantitative RT-PCR, which was performed in triplicate for each sample. Data were normalized to UBC (a house-keeping gene) and are shown as means ± SD. **(D)** 2-d LPS-stimulated XBP1^KO^ and S729A/XBP1^KO^ B cells were further stimulated with LPS for an additional 8, 16, and 24 h. The levels of μS mRNA were measured by quantitative RT-PCR. **(E and F)** WT, XBP1^KO^, DKO, and S729A/XBP1^KO^ B cells were stimulated with LPS for 3 d. The mRNA levels of Tapbp1 (E) and Hgsnat (F) were measured by quantitative RT-PCR. **(G–M)** IRE1^−/−^ MEFs were transfected with pcDNA3.1 plasmids carrying full-length mouse IRE1 cDNA (WT) or carrying the S729D or S729E mutation. The mRNA levels of Ergic3 (G), Scara3 (H), pdgfrb (I), Col6a1 (J), Tapbp1 (K), Bloc1S1 (L), and Hgsnat (M) were measured by quantitative RT-PCR 48 h after transfection. Data are shown as means ± SD. All quantitative RT-PCR data shown are representative of three independent experiments.

XBP1 deficiency leads to the up-regulated levels of IRE1 and RIDD in liver, pancreas, and B cells (Lee et al., 2011; Hur et al., 2012; So et al., 2012; Benhamron et al., 2014). In XBP1-deficient B cells, μS mRNA is destroyed by RIDD, which leads to significantly decreased levels of sIgM (Benhamron et al., 2014). To test the potential contribution of S729 phosphorylation of IRE1 to RIDD, we crossed S729A mice with B cell–specific XBP1^KO^ (CD19Cre/XBP1^f/f^) mice to generate S729A/XBP1^KO^ mice. As critical controls, we generated B cell–specific IRE1^KO^ mice by crossing IRE1^f/f^ mice (Zhang et al., 2011) with CD19Cre mice. CD19Cre/IRE1^f/f^ mice were further crossed with XBP1^f/f^ mice to generate B cell–specific IRE1^KO^/XBP1^KO^ (CD19Cre/IRE1^f/f^/XBP1^f/f^) mice. We purified B cells from spleens of WT, XBP1^KO^, IRE1^KO^, IRE1^KO^/XBP1^KO^, S729A, and S729A/XBP1^KO^ mice and stimulated those B cells with LPS for 3 d (Fig. 7, A and B). Upon LPS stimulation, IRE1^KO^ and IRE1^KO^/XBP1^KO^ B cells produced a 115-kD ΔIRE1 protein, which lacked 100 amino acids (from aa 652 to aa 751) in the kinase domain (Fig. 7 A). Consistent with previous studies, the lack of XBP1s caused normal and leukemic B cells to produce up-regulated levels of IRE1 (Fig. 7 A; Hu et al., 2009a; Tang et al., 2014), contributing to the increased RIDD (Benhamron et al., 2014) and decreased protein levels of the μS chains (Fig. 7 A). The lack of functional IRE1 in IRE1^KO^ and IRE1^KO^/XBP1^KO^ B cells abolished RIDD, allowing B cells to continue synthesizing μS chains (Fig. 7 A). LPS-stimulated S729A B cells produced XBP1s and synthesized μS chains, and LPS-stimulated S729A/XBP1^KO^ B cells also up-regulated the S729-mutated IRE1 protein (Fig. 7 B). Notably, the S729A mutation, similar to IRE1^KO^, abolished RIDD, resulting in increased expression of μS mRNA and μS chains in S729A/XBP1^KO^ B cells (Fig. 7, A–D). In addition, the S729A mutation and IRE1^KO^ almost completely blocked RIDD of Tapbp1 and Hgsnat mRNAs in XBP1^KO^ B cells (Fig. 7, E and F). Although both S729A and IRE1^KO^ abolished RIDD to allow for the recovery of μS chains, we did not observe a complete recovery of μS mRNA levels, suggesting that RIDD was not the only mechanism that caused the degradation of μS mRNA in XBP1-deficient B cells (Fig. 7 C).

To further confirm that S729 phosphorylation played a role in regulating RIDD, we transfected IRE1^−/−^ mouse embryonic fibroblasts (MEFs) with IRE1 carrying phosphomimetic mutations (S729D and S729E) and showed that these cells expressed significantly less mRNA levels of RIDD substrates when compared with those transfected with WT IRE1 (Fig. 7, G–M).

### SubAB induced RIDD in B cells

**Figure 8.**
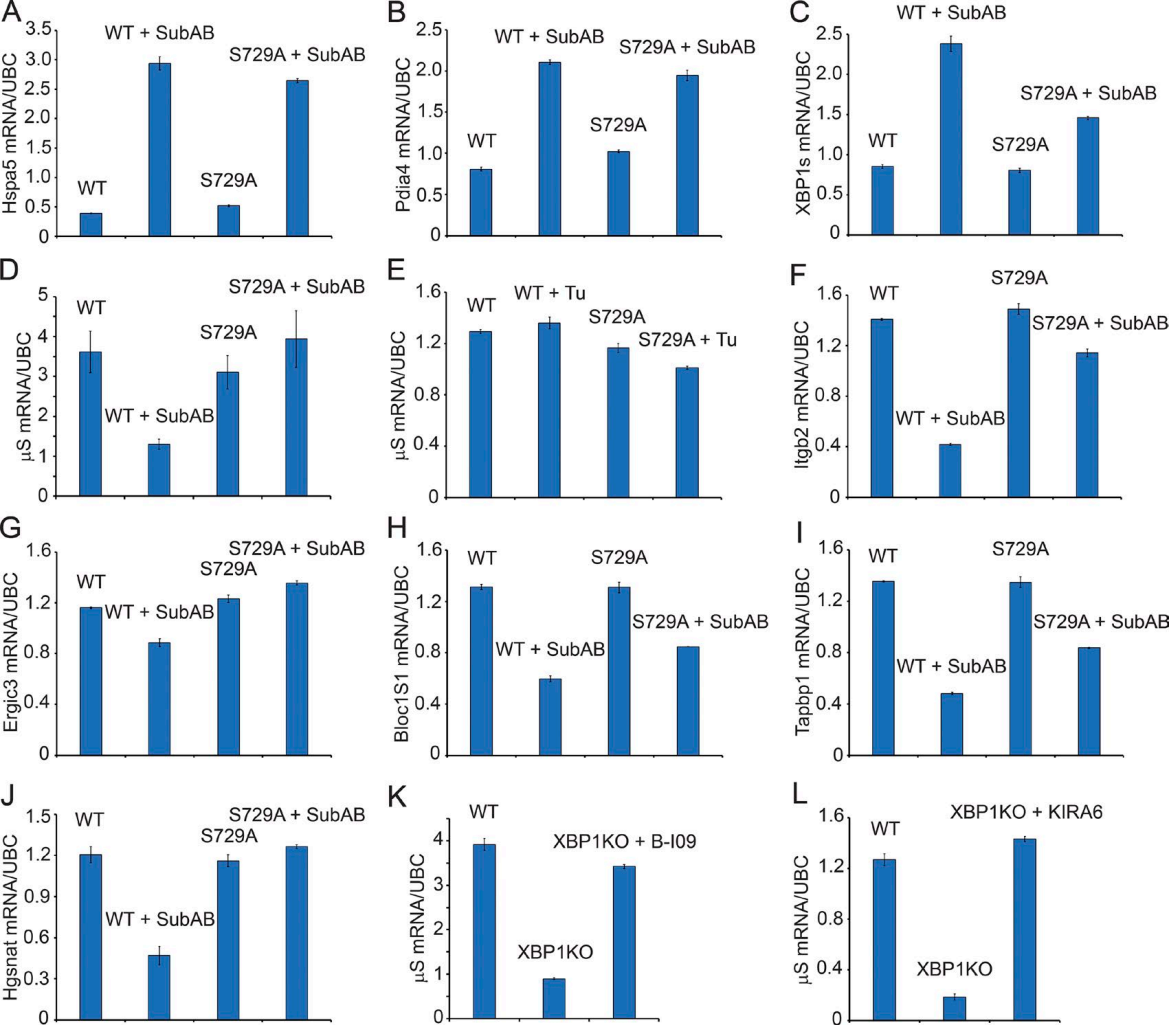
**SubAB induces RIDD in B cells. (A–D)** 2-d LPS-stimulated WT and S729A B cells were exposed to 1 nM SubAB for 24 h. The mRNA levels of Hspa5 (A), Pdia4 (B), XBP1s (C), and μS (D) were measured by quantitative RT-PCR. **(E)** 2-d LPS-stimulated WT and S729A B cells were exposed to 5 µg/ml Tu for 24 h. The μS mRNA levels were measured by quantitative RT-PCR. **(F–J)** 2-d LPS-stimulated WT and S729A B cells were exposed to 1 nM SubAB for 24 h. The mRNA levels of the indicated genes were measured by quantitative RT-PCR. **(K and L)** Naive B cells from WT and XBP1^KO^ mice were stimulated with LPS for 3 d. Some XBP1^KO^ B cells were incubated with 20 µM B-I09 (K) or 5 µM KIRA6 (L) in the last 24 h of LPS stimulation. The μS mRNA levels were measured by quantitative RT-PCR. Data are shown as means ± SD. Quantitative RT-PCR data are representative of three independent experiments.

Because SubAB triggered S729 phosphorylation effectively (Fig. 2, A, D, and E), we tested whether SubAB could induce RIDD in B cells. We exposed LPS-stimulated WT and S729A B cells to SubAB for 24 h and confirmed that SubAB induced these B cells to express high levels of Hspa5 (BiP) and Pdia4 (protein disulfide isomerase A4) mRNAs (Fig. 8, A and B). Congruent with our flow cytofluorometry data (Figs. 6 C and S5 B), SubAB induced LPS-stimulated S729A B cells to express less XBP1s mRNA than LPS-stimulated WT B cells (Fig. 8 C). SubAB indeed induced RIDD as shown by the decreased levels of μS mRNA in WT but not S729A B cells upon SubAB treatment (Fig. 8 D). Because Tu did not cause phosphorylation of S729 (Figs. 2 A and S1, C and D), we also found that it did not induce RIDD of μS mRNA in B cells (Fig. 8 E). We further investigated the response of other RIDD substrates in SubAB-treated WT and S729A B cells (Fig. 8, F–J). Similar to μS mRNA results (Fig. 8 D), SubAB induced RIDD of these mRNA substrates in WT B cells but less so in S729A B cells (Fig. 8, F–J).

### B-I09 and KIRA6 inhibited RIDD in XBP1-deficient B cells

B-I09 inhibits IRE1 from splicing the XBP1 mRNA (Figs. 2 A, 4 C, and 6 A; Tang et al., 2014). We tested whether B-I09 could also inhibit RIDD. B-I09 efficiently blocked the degradation of μS mRNA in XBP1-deficient B cells (Fig. 8 K), suggesting that B-I09 was also an inhibitor of RIDD. KIRA6 has a better activity in inhibiting IRE1 from cleaving insulin mRNA (an RIDD substrate) than from splicing the XBP1 mRNA (Ghosh et al., 2014). Intrigued by our results showing that KIRA6 inhibited S729 phosphorylation and had little effect in suppressing the expression of XBP1s (Figs. 2 F and S2, A–D), we hypothesized that KIRA6 could inhibit the degradation of μS mRNA in XBP1-deficient B cells and showed that this was indeed the case (Fig. 8 L).

### Immunized S729A mice produced increased amounts of IgM and IgG2b in the blood

**Figure 9.**
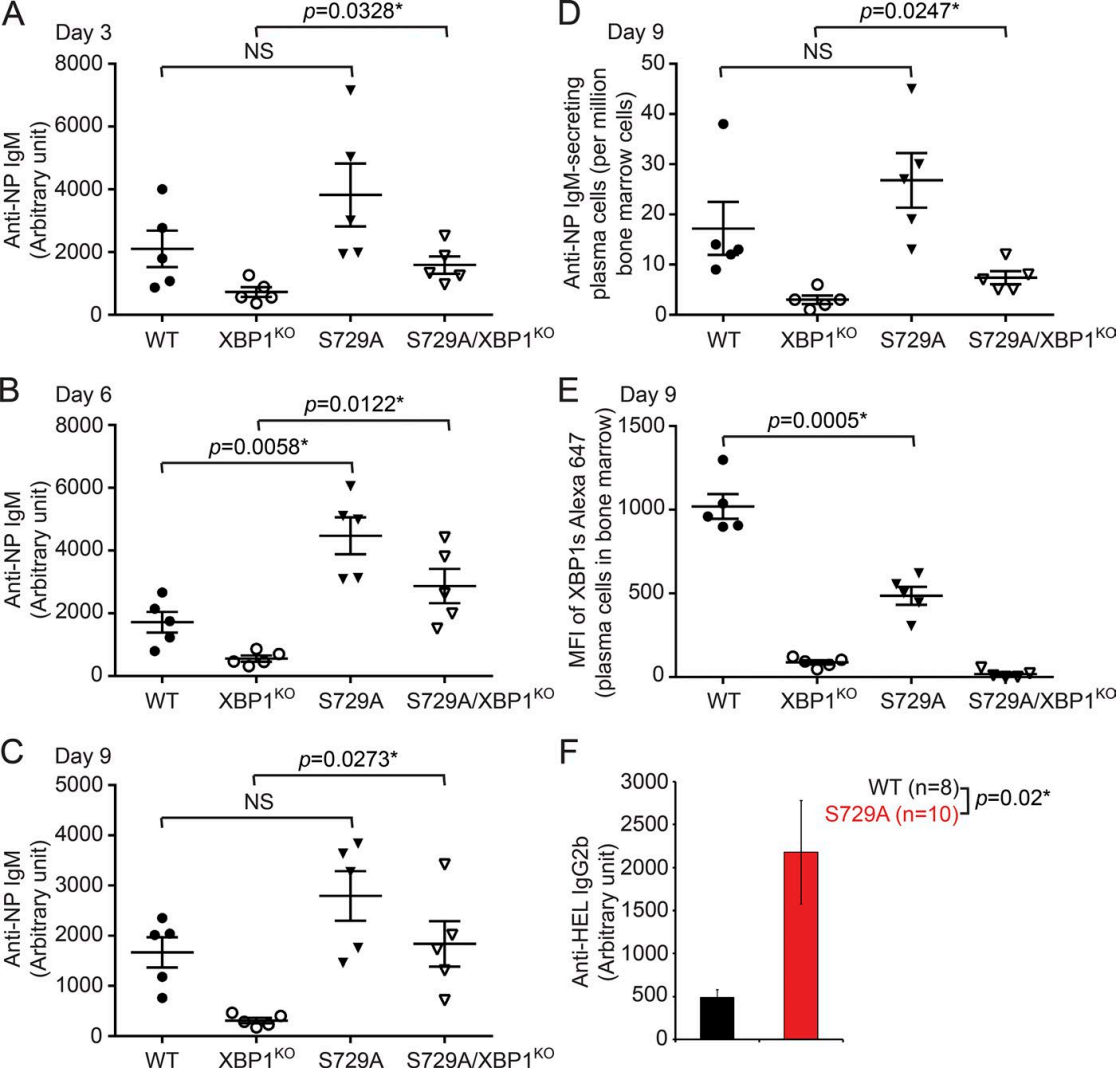
**Immunized S729A mice produce increased serum levels of IgM and IgG2b. (A–C)** Serum levels of anti–NP IgM in WT, XBP1^KO^, S729A, and S729A/XBP1^KO^ mice (*n* = 5 per group) were determined by ELISA on day 3 (A), day 6 (B), and day 9 (C) after immunization with NP-Ficoll on day 0. **(D and E)** On day 9, NP-Ficoll–immunized mice were sacrificed to analyze for anti–NP IgM–secreting plasma cells in the bone marrow by ELISPOT (D). Bone marrow cells isolated from immunized mice were stained with CD138-PE, B220-BV605, and XBP1s–Alexa Fluor 647. Gated CD138^+^ populations were analyzed for the expression of XBP1s (E). **(F)** Serum levels of anti–HEL IgG2b in WT and S729A mice immunized with HEL/CFA followed by 3 boosts with HEL/IFA were determined by ELISA. Data are shown as means ± SEM.

To explore the role of S729 in vivo, we immunized WT, XBP1^KO^, S729A, and S729A/XBP1^KO^ mice with NP-Ficoll to interrogate B cell–intrinsic role of S729. B cells respond to such immunization by producing NP-specific IgM. We analyzed NP-specific IgM levels in sera at 3, 6, and 9 d after immunization (Fig. 9, A–C) and detected significantly increased IgM levels in S729A mice on day 6, when compared with WT mice (Fig. 9 B). When we compared immunized S729A/XBP1^KO^ with XBP1^KO^ mice, we detected consistently increased levels of IgM and increased frequencies of NP-specific plasma cells in S729A/XBP1^KO^ mice (Fig. 9, A–D). Although we did not detect a significant difference in NP-specific plasma cell frequencies between immunized WT and S729A mice (Fig. 9 D), the expression levels of XBP1s were significantly lower in plasma cells of immunized S729A mice (Fig. 9 E). Such data support the hypothesis that S729A mutation compromises IRE1’s function in splicing XBP1 mRNA, leading to decreased levels of XBP1s, and in cleaving μS mRNA, leading to increased levels of IgM.

Although we did not detect significantly increased anti–HEL IgG or IgG1 in S729A mice repeatedly immunized with HEL (Fig. 4, I and J), such immunization allowed further class-switch recombination. As demonstrated, the γ2b mRNA is an RIDD substrate (Benhamron et al., 2014). We examined anti–HEL IgG2b levels in repeated immunized WT and S729A mice and found significantly increased serum levels of IgG2b in immunized S729A mice (Fig. 9 F), supporting the idea that plasma cells of immunized S729A mice acquired defective RIDD.

## Discussion

**Figure 10.**
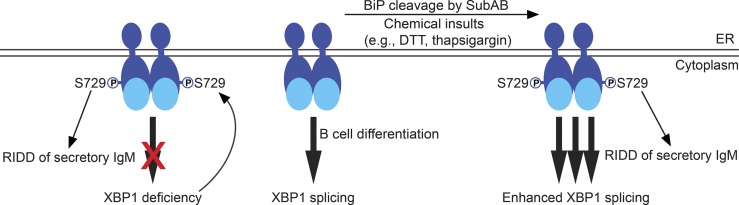
**A model depicting S729 phosphorylation of IRE1 enhancing the expression of XBP1s and activating RIDD in B cells.**

The kinase domain of IRE1 is critical for the RNase activity of IRE1 as demonstrated by our data showing that missing aa 652–751 in the kinase domain completely blocks the expression of XBP1s in LPS-stimulated B cells (Fig. 7 A); however, IRE1 carrying the S729A mutation can still express XBP1s (Figs. 5 A and 7 B). In XBP1^KO^ B cells, up-regulated IRE1 undergoes phosphorylation at S729 (Fig. 1, F and H) and contributes to RIDD (Fig. 10). This is supported by the results showing that the S729A mutation can inhibit IRE1 from cleaving RIDD substrates in XBP1^KO^ B cells (Fig. 7, B–F). In contrast with inhibiting RIDD in XBP1^KO^ B cells by deleting the RNase activity of IRE1 (Benhamron et al., 2014), our data showed that deleting the kinase function or mutating S729 in the kinase domain of IRE1 was sufficient to block RIDD in XBP1^KO^ B cells (Fig. 7, A–F). Distinct from the two different B cell–specific IRE1^KO^ mouse models in which both XBP1 splicing and RIDD are blocked (Fig. 7, A, C, E, and F; Benhamron et al., 2014), the S729A mutation only blocks RIDD (Fig. 7, B–F), highlighting the critical function of S729 in regulating RIDD in B cells. When we immunized WT and S729A mice with a T-independent antigen (NP-Ficoll), we detected increased levels of anti–NP IgM in S729A mice (Fig. 9, A–C). In mice repeatedly immunized with a T-dependent antigen (HEL), we also detected significantly increased IgG2b levels in S729A mice (Fig. 9 F). Such data suggest that S729 phosphorylation on IRE1 is critical for plasma cells to curtail the production of IgM and certain Ig isotypes such as IgG2b in response to immunization.

By transfecting insect cells with the cytoplasmic domain (aa 547–977) of human IRE1, IRE1 was shown to be phosphorylated at S551, S562, S724, S726, S729, and T973 (Prischi et al., 2014). Although S724, S726, and S729 are located in the activation loop of human IRE1 and are hypothesized to be critical for regulating the RNase activity of IRE1, IRE1 carrying S724A/S726A/S729A triple mutations can still robustly splice the XBP1 mRNA (Prischi et al., 2014), suggesting that phosphorylation of mammalian IRE1 at the activation loop is not required for splicing of the XBP1 mRNA. Yeast Ire1 carrying all 5 phosphorylation mutations in the activation loop can still splice HAC1 mRNA (Armstrong et al., 2017). When HT1080 cells were transfected with the S724A/S726A double mutant or the S724A/S726A/S729A triple mutant, the two mutants showed similar effects in splicing XBP1 mRNA, leading to the conclusion that the RNase activity of human IRE1 can only be enhanced by phosphorylation of S724 and S726 (Prischi et al., 2014). IRE1 in LPS-stimulated B cells does not require phosphorylation to activate XBP1 (Fig. 1, H and J). When compared with LPS-stimulated WT B cells, LPS-stimulated S729A B cells do not exhibit changes in the mRNA levels of XBP1s and RIDD substrates (Fig. 8, C–J), further suggesting that IRE1 does not require phosphorylation at S729 to splice or cleave mRNAs in LPS-stimulated WT B cells. Although Tg induces phosphorylation of IRE1 only at S729 in LPS-stimulated B cells, SubAB and DTT can induce phosphorylation at S729 and other sites in these cells (Fig. 6 E). However, S729A mutation completely blocks IRE1’s ability to further up-regulate XBP1s to respond to any of these ER stress conditions (Fig. 6). We propose that S729 may be the first residue undergoing phosphorylation in the activation loop of IRE1 in LPS-stimulated WT B cells in response to SubAB treatment as indicated by no IRE1 band shift in LPS-stimulated S729A B cells at the early time points of the pulse-chase experiment (Fig. 4 A). In addition, the slightly decreased levels of XBP1s in LPS-stimulated S729A B cells (Fig. 5, A, C, and D; and Figs. 6 D and 7 B) suggest that XBP1s may be directly or indirectly stabilized by IRE1 and that the replacement of the polar S729 residue by a hydrophobic alanine may compromise such a stabilizing effect.

Phosphorylation at S729 of IRE1 occurs in B cells lacking XBP1s or exposed to SubAB (Fig. 1, F, H, and I). Both XBP1 deficiency and SubAB treatment lead to degradation of RIDD substrates in B cells, and such degradation can be blocked by S729A mutation (Fig. 7, B–F; and Fig. 8, D and F–J). Tu does not induce phosphorylation of S729 on IRE1, although it can induce the expression of XBP1s by activating other phosphorylation sites (Fig. S1, C, D, and J). As expected, Tu fails to induce RIDD of μS mRNA in B cells (Fig. 8 E). Together with data showing that RIDD is readily observed in IRE1^−/−^ MEFs transfected with phosphomimetic mutants of IRE1 (Fig. 7, G–M), phosphorylation at S729 is indispensable for RIDD.

In addition to regulating RIDD, phosphorylation at S729 can enhance the RNase activity of IRE1 in splicing XBP1 (Fig. 10). Although XBP1 splicing can occur in LPS-stimulated S729A B cells (Fig. 5, A and C; and Figs. 6 D and 7 B), these B cells fail miserably in their response to secondary ER stress insults (Fig. 6, A–D; and Fig. S5). We propose that S729 phosphorylation triggers enhanced XBP1 splicing and RIDD, which can together relieve ER stress in B cells by synthesizing more lipids and chaperones to increase the folding capacity of the ER via enhanced XBP1 splicing and by reducing the production of μS chains and other proteins via RIDD. In B cells that lack XBP1s, S729 phosphorylation allows activation of RIDD to reduce the amounts of newly synthesized unfolded μS chains to enter the ER, thus protecting XBP1-deficient B cells from proteotoxicity (Fig. 10). We showed previously that SubAB can cleave BiP in B cells, and the resultant C-terminal substrate-binding domain of BiP can efficiently sequester the κ light chain together with μ heavy chains inside B cells (Hu et al., 2009b); here, SubAB-induced S729 phosphorylation can activate RIDD to reduce the production of secretory μ chains (Fig. 8 D), representing another tactic of Shiga-toxigenic *E. coli* in dismantling the host’s B cell function.

The IRE1 RNase inhibitor B-I09 inhibits IRE1 from splicing XBP1 and engaging RIDD (Figs. 2 A, 4 C, 6 A, and 8 K; Tang et al., 2014), supporting that the “same” RNase domain of IRE1 splices XBP1 mRNA and cleaves μS mRNA. We hypothesize that phosphorylation at S729 might allow dimeric or oligomeric IRE1 to interact with other proteins in the UPRosome (Hetz et al., 2011; Woehlbier and Hetz, 2011), resulting in further assembly of its RNase domains to assume a more “relaxed and promiscuous” conformation to enhance XBP1 splicing and cleave RIDD substrates. Because S729 phosphorylation of IRE1 is critical in regulating RIDD, small molecules that specifically inhibit S729 phosphorylation can be useful tools in interrogating the biology of RIDD in human diseases. KIRA6 is one such inhibitor because it rapidly inhibits S729 phosphorylation of IRE1 and suppresses RIDD without affecting the levels of XBP1s (Figs. 2 F, 8 L, and S2, A–D).

## Materials and methods

### Generation and maintenance of the S729A knock-in mouse model

10 µg of the targeting vectors were transfected by electroporation of C57BL/6 (B6) embryonic stem (ES) cells. After selection with the G418 antibiotic, surviving clones were expanded for PCR analysis to identify recombinant ES clones. Primers designed within the Neo cassette (forward) and downstream of the short homology arm (reverse) were used for screening by PCR to identify positive clones, which were then selected for expansion and reconfirmed for short homology arm integration. Sequencing was performed on purified PCR DNA to confirm the presence of the point mutation. Secondary confirmation of positive clones identified by PCR was performed using Southern blotting analysis to confirm the integration of 3′ and 5′ homology arms. In brief, restriction-digested DNA was electrophoretically separated on a 0.8% agarose gel, transferred to a nylon membrane, and hybridized with a probe targeted against the Neo cassette. The probe includes part of the Neo sequence as well as the mouse genomic sequence and hybridizes to the Neo cassette and the genomic sequence to generate two bands (targeted and WT) on the blot. DNA from the B6 mouse strain was used as the WT control. Each clonal cell culture was sampled before injection and had an equal passage number to the injected cells. For each clone, 20 metaphase spreads were analyzed, and the percent euploidy in each culture was calculated following the Cold Spring Harbor Laboratory’s chromosome counting protocol of ignoring metaphase spreads with <39 chromosomes. All clones passed the required 70% euploid cutoff. Targeted ES cells were microinjected into BALB/c blastocysts. Resulting chimeras with a high percentage black-coat coloring were mated to C57BL/6 flippase (FLP) mice to remove the Neo cassette, which is flanked by FLP recognition target sites. Tail DNA samples from pups with black coat coloring were screened for the deletion of the Neo cassette by PCR using designed primers. Again, sequencing was performed on purified PCR DNA to confirm the presence of the desired point mutation. We also confirmed the absence of the FLP transgene and reconfirmed the integration of the short homology arm by PCR using specific primers. The mice, confirmed for somatic Neo deletion, were mated to WT B6 mice to generate germline Neo-deleted mice, which were again genotyped for the presence of Neo deletion and the absence of the FLP transgene. These germline Neo-deleted mice were mated to each other to generate homozygous mice. Homozygous male S729A mice must be crossed with heterozygous female S729A mice to maintain the S729A mouse colony. We set up >12 breeding pairs using homozygous S729A female mice, but these homozygous S729A female mice never gave birth to pups, consistent with a previous study showing a critical role for IRE1 in the placenta during pregnancy (Iwawaki et al., 2009). Histological examinations of the female reproductive organs in S729A mice showed the normal ovarian cycle with follicles of every stage identified. The S729A male and female pups are born at a normal Mendelian ratio. In the S729A mouse colony, ∼50% pups carry homozygous S729A alleles.

### Mice

The S729A knock-in mice, together with XBP1^f/f^, CD19Cre/XBP1^f/f^, IRE1^f/f^ (provided by D. Fang, Northwestern University, Chicago, IL), CD19Cre/IRE1^f/f^, IRE1^f/f^/XBP1^f/f^, CD19Cre/IRE1^f/f^/XBP1^f/f^, S729A/XBP1^f/f^, and S729A/CD19Cre/XBP1^f/f^ mice were maintained at our animal facility strictly following the guidelines provided by the Wistar Institute Committee on Animal Care.

### Study approval

All experiments involving the use of mice were performed following protocols approved by the Institutional Animal Care and Use Committee at the Wistar Institute.

### Purification of mouse B cells

Splenocytes were obtained from mice by mashing the spleens through cell strainers followed by RBC lysis (Sigma-Aldrich). Mouse B cells were purified from mouse spleens by negative selection using CD43 (Ly48) or pan-B magnetic beads (Miltenyi Biotec), according to the manufacturer’s instructions.

### Mass spectrometry

Protein bands were stained with Coomassie brilliant blue G-250, excised, reduced, and alkylated. In-gel proteolytic digestion with trypsin, chymotrypsin, or both was used to prepare samples for peptide sequencing with reverse-phase LC-MS/MS using Dionex ultraperformance liquid chromatography (Thermo Fisher Scientific) interfaced with an electrospray hybrid, linear-ion trap–orbital ion trap (LTQ Orbitrap; Thermo Fisher Scientific) mass spectrometer. Protonated peptide molecules were measured in positive ion mode in the survey scan (MS1), before selection of the top seven ion signals for tandem mass spectrometry. Fragment ion mass spectra obtained from LC-MS/MS were submitted to SEQUEST (Thermo Fisher Scientific) and MASCOT software (Matrix Science) searches for entries of the appropriate species in the UniProt database. In addition to mass tolerances appropriate to the instrument, two missed cleavages and variable modifications (Ser/Thr/Tyr phosphorylation and Met oxidation) were included in the search parameters. Matched sequences were summarized in Scaffold software (Proteome Software); single peptide matches for protein identifications and all phosphorylated peptides were further verified by manual inspection of the tandem mass spectrometry spectra and relatively quantified using XICs.

### Flow cytometric analysis

Single-cell suspensions from spleens, bone marrow, or peripheral lymph nodes were blocked for 30 min using FBS. Cell-surface staining was achieved by incubating cells at 4°C for 30 min with fluorescence-conjugated anti–mouse antibodies (clone; source): XBP1s–Alexa Fluor 647 (Q3-695; BD), XBP1s-phycoerythrin (PE; Q3-695; BD), B220–Alexa Fluor 488 (RA3-6B2; BioLegend), B220-BV605 (RA3-6B2; BioLegend), CD43-PE (eBioR2/60; eBioscience), CD19–Alexa Fluor 647 (6D5; BioLegend), IgM-PE-Cy7 (RMM-1; BioLegend), IgD-FITC (11-26c.2a; BioLegend), GL7-PE (GL7; BioLegend), AA4.1-PE-Cy7 (AA4.1; BioLegend), CD1d-PerCP-Cy5.5 (1B1; BioLegend), CD23-FITC (B3B4; BioLegend), CD3-APC-Cy7 (145-2C11; BioLegend), CD4-BV605 (RM4-5; BioLegend), CD8α-PE-Cy7 (53–6.7; BioLegend), and CD138-PE (281–2; BioLegend). Viability staining was accomplished using DAPI exclusion during acquisition. Acquisition of B, T, and dendritic cell populations was performed on an LSRII cytometer (BD) harboring a custom configuration for the Wistar Institute. Cytometry data were analyzed using FlowJo software (7.6.1; Tree Star Inc.).

### Antibodies and reagents

Rabbit polyclonal antibodies against recombinant mouse IRE1 (aa 21–445) and a phospho-S729 peptide were generated and affinity purified. Polyclonal antibodies against BiP/GRP78 and PDI were also generated in rabbits. The following antibodies were obtained commercially: IRE1 (Cell Signaling Technology), XBP1s (Cell Signaling Technology), GRP94 (Stressgen), p97 (Fitzgerald Industries), μ (SouthernBiotech), κ (SouthernBiotech), phospho-ERK1/2 (Cell Signaling Technology), ERK1/2 (Cell Signaling Technology), phospho–nuclear factor κ–light chain enhancer of activated B cells (NF-κB) p105 (Cell Signaling Technology), phospho–NF-κB p65 (Cell Signaling Technology), and caspase 3 (Cell Signaling Technology). LPS (Sigma-Aldrich), DTT (Sigma-Aldrich), BFA (Cell Signaling Technology), CpG-1826 oligodeoxynucleotides (TIB-Molbiol), Tu (Enzo Life Sciences), Tg (Enzo Life Sciences), KIRA6 (EMD Millipore), staurosporine (EMD Millipore), imatinib (EMD Millipore), sunitinib (Sigma-Aldrich), MG-132 (Enzo Life Sciences), Z-VAD-FMK (Enzo Life Sciences), chloroquine (Cell Signaling Technology), and NH_4_Cl (Sigma-Aldrich) were also purchased from commercial sources. Native and mutant SubAB were purified as described previously (Paton et al., 2004; Talbot et al., 2005). Shiga toxin 1, Shiga toxin 2, cholera toxin, and pertussis toxin were purchased from the List Biological Laboratories. We developed and chemically synthesized the IRE1 RNase inhibitor B-I09 (Tang et al., 2014).

### Cell culture

Purified naive mouse B cells, mouse 5TGM1 myeloma cells (provided by L.A. Hazlehurst, West Virginia University, Morgantown, WV), mouse A20 B cell lymphoma cells (ATCC), and human H929 myeloma cells (ATCC) were cultured in the RPMI-1640 medium (Gibco) supplemented with 10% heat-inactivated FBS, 2 mM l-glutamine, 100 U/ml penicillin G sodium, 100 µg/ml streptomycin sulfate, 1 mM sodium pyruvate, 0.1 mM nonessential amino acids, and 0.1 mM β-mercaptoethanol (β-ME). 5TGM1 cells were tested for the secretion of Ig and the surface expression of plasma cell marker CD138 every 6 mo. All cell lines were negative for mycoplasma contamination.

### Site-directed mutagenesis and transfection

IRE1^−/−^ MEFs were provided by D. Ron (University of Cambridge, Cambridge, England, UK) and cultured in DMEM supplemented with 10% FBS. Full-length mouse IRE1 cDNA was cloned into pcDNA3.1^+^ plasmid between EcoRI and NotI restriction sites. Mutagenesis was performed according to the manufacturer's instructions using the QuikChange II XL site-directed mutagenesis kit (Agilent Technologies). The sense oligonucleotides used for S729D and S729E were as follows, with the altered codons underlined: 5′-GGCACAGTTTCAGCCGCCGTGATGGGGTACCTGGC-3′ (S729D) and 5′-GGCACAGTTTCAGCCGCCGTGAGGGGGTACCTGGC-3′ (S729E). IRE1^−/−^ MEFs were transfected using Lipofectamine 3000 reagent (Thermo Fisher Scientific) according to the manufacturer’s instructions.

### Protein isolation, immunoprecipitation, dephosphorylation, and immunoblotting

Cells were lysed in radioimmunoprecipitation assay buffer (10 mM Tris-HCl, pH 7.4, 150 mM NaCl, 1% NP-40, 0.5% sodium deoxycholate, 0.1% SDS, and 1 mM EDTA) supplemented with protease inhibitors (Roche) and phosphatase inhibitors. Protein concentrations were determined by bicinchoninic acid assays (Thermo Fisher Scientific). IRE1 was immunoprecipitated with an anti–mouse IRE1 antibody and protein G–Sepharose beads (Sigma-Aldrich). Bead-bound IRE1 was dephosphorylated using λPPase (New England Biolabs, Inc.) or CIP (New England Biolabs, Inc.). Proteins were boiled in SDS-PAGE sample buffer (62.5 mM Tris-HCl, pH 6.8, 2% SDS, 10% glycerol, and 0.1% bromophenol blue) with β-ME, analyzed by SDS-PAGE, and transferred to nitrocellulose membranes, which were then blocked in 5% nonfat milk (wt/vol in PBS) and immunoblotted with the indicated primary antibodies and appropriate HRP-conjugated secondary antibodies. Immunoblots were developed with Western Lightning chemiluminescence reagent (Perkin-Elmer).

### Pulse-chase experiments, immunoprecipitation, and protein dephosphorylation

Cells were starved in methionine- and cysteine-free media containing dialyzed FBS for 1 h and pulse-labeled with 250 µCi/ml [^35^S]methionine and [^35^S]cysteine (Perkin-Elmer) for the indicated times. After labeling, cells were incubated in the chase medium containing unlabeled methionine (2.5 mM) and cysteine (0.5 mM). At the end of each chase interval, cells were lysed in radioimmunoprecipitation assay buffer containing protease inhibitors. Precleared lysates were incubated with an anti–mouse IRE1 antibody and protein G–Sepharose beads. To enzymatically remove the phosphate group, bead-bound IRE1 was incubated with λPPase. Samples were boiled in SDS-PAGE sample buffer (62.5 mM Tris-HCl, pH 6.8, 2% SDS, 10% glycerol, and 0.1% bromophenol blue) with β-ME, analyzed by SDS-PAGE, and visualized by autoradiography.

### RT-PCR

Total RNA was isolated with TRIzol reagent (Invitrogen). cDNA was synthesized from RNA with Superscript II reverse transcription (Invitrogen). The following sets of primers were used together with Platinum Taq DNA polymerase (Invitrogen) in PCR to detect the expression of mouse μS, 5′-CACACTGTACAATGTCTCCCT-3′ and 5′-AAAATGCAACATCTCACTCTG-3′; mouse UBC, 5′-CAGCCGTATATCTTCCCAGACT-3′ and 5′-CTCAGAGGGATGCCAGTAATCTA-3′; mouse XBP1s, 5′-GTCCATGGGAAGATGTTCTGG-3′ and 5′-CTGAGTCCGAATCAGGTGCAG-3′; mouse Hspa5, 5′-TGTCTTCTCAGCATCAAGCAAGG-3′ and 5′-CCAACACTTTCTGGACAGGCTT-3′; mouse Pdia4, 5′-GGGCTCTTTCAGGGAGATGG-3′ and 5′-GGGAGACTTTCAGGAACTTGGC-3′; mouse Itgb2, 5′-CTTTCCGAGAGCAACATCCAGC-3′ and 5′-GTTGCTGGAGTCGTCAGACAGT-3′; mouse Ergic3, 5′-GTTCAAGAAACGACTAGACAAGGA-3′ and 5′-ACCTCGACTTTCCCAAGCTC-3′; mouse Bloc1S1, 5′-GAGGAGAGAAGCTATCGCTGCA-3′ and 5′-CTGGACCTGTAGAGTCTTCACC-3′; mouse Tapbp1, 5′-ACCATTCCCAGGAACTCAAA-3′ and 5′-GAGAAGAAGGCTGTTGTTCTGG-3′; mouse Hgsnat, 5′-TCTCCGCTTTCTCCATTTTG-3′ and 5′-CGCATACACGTGGAAAGTCA-3′; mouse Scara3, 5′-TGCATGGATACTGACCCTGA-3′ and 5′-GCCGTGTTACCAGCTTCTTC-3′; mouse Col6a1, 5′-TGCTCAACATGAAGCAGACC-3′ and 5′-TTGAGGGAGAAAGCTCTGGA-3′; and mouse Pdgfrb, 5′-AACCCCCTTACAGCTGTCCT-3′ and 5′-TAATCCCGTCAGCATCTTCC-3′.

### Immunization

Mice were intraperitoneally immunized with HEL (100 µg/mouse; Sigma-Aldrich) mixed in CFA or IFA (Sigma-Aldrich) or with NP_55_-aminoethylcarboxymethyl-Ficoll (40 µg/mouse; LGC Biosearch Technologies) in PBS.

### ELISA and enzyme-linked immunospot (ELISPOT) assay

For ELISA, plates were coated with HEL or NP_23_-BSA. ELISA analyses of mouse IgM, IgG, IgG1, and IgG2b in immunized mouse sera were achieved with HRP-conjugated secondary antibodies against each mouse antibody isotype (SouthernBiotech) and 3,3′,5,5′-tetramethylbenzidine liquid substrate system (Sigma-Aldrich). For ELISPOT assay, MultiScreen-IP filter plates (EMD Millipore) were coated with NP_23_-BSA. Bone marrow cells were serially diluted across the plate and then incubated for 16 h at 37°C. HRP-conjugated goat anti–mouse IgM antibodies (SouthernBiotech) diluted in blocking buffer (10% FBS in PBS) were added. Spots were detected with the 3,3′,5,5′-tetramethylbenzidine substrate for ELISPOT (Mabtech) and scanned and counted with an ImmunoSpot Analyzer (Cellular Technology Ltd.).

### Statistics

For comparison of percentages of cell populations among experimental groups, data were graphed as means ± SEM. Statistical significance (P < 0.05) was determined by Student’s *t* test.

### Online supplemental material

Fig. S1 shows that Tg, but not Tu, BFA, or MG132, induces IRE1 to undergo phosphorylation at S729. Fig. S2 shows that KIRA6 rapidly suppresses SubAB-induced S729 phosphorylation of IRE1 and that Shiga toxins, cholera toxin, and pertussis toxin do not induce S729 phosphorylation even when used at high concentrations. Fig. S3 documents the generation of the S729A mouse model and shows that B cells develop normally in the bone marrow of unimmunized S729A mice and that after immunization, the percentages of pro– and mature B cells increase in the bone marrow of S729A mice. Fig. S4 documents normal B and T cell percentages in the spleens and lymph nodes of unimmunized S729A mice and shows that after immunization, the percentages of CD19^+^ B cells decrease in the lymph nodes, whereas the percentages of CD4^+^ and CD8^+^ T cells increase in the spleens and lymph nodes of S729A mice. Fig. S5 shows that LPS-stimulated B cells from S729A mice fail to respond to Tg or SubAB by enhancing the expression of XBP1s.

## Supplementary Material

Supplemental Materials (PDF)
