# Supplementary material for: Phosphorylation of IRE1 at S729 regulates RIDD in B cells and antibody production after immunization
Source: J Cell Biol. 2018 May 7;217(5):1739–55. doi: 10.1083/jcb.201709137 (PMC5940306; doi:10.1083/jcb.201709137)
Supplement: Supplemental Materials (PDF) [file JCB_201709137_sm.pdf]

Supplemental material

Tang et al., https://doi.org/10.1083/jcb.201709137

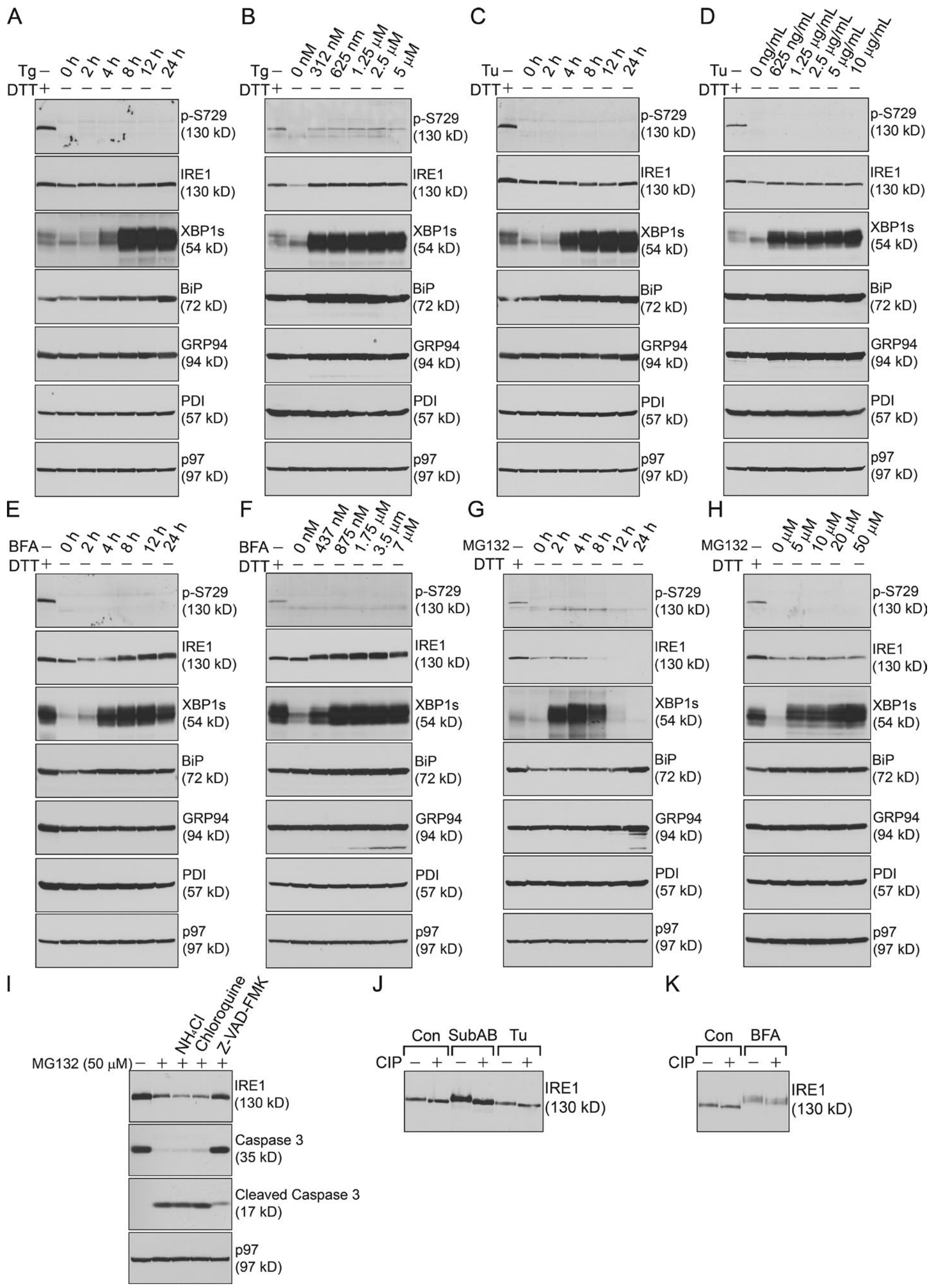

Figure S1. **Tg, but not Tu, BFA, or MG132, induces IRE1 to undergo phosphorylation at S729. (A)** 5TGM1 cells were treated with 5 mM DTT for 3 h or with 2.5 µM Tg for a course of 24 h and immunoblotted for the indicated proteins. **(B)** 5TGM1 cells were treated with 5 mM DTT for 3 h or with Tg for 24 h using increasing concentrations and were immunoblotted for the indicated proteins. **(C)** 5TGM1 cells were treated with 5 mM DTT for 3 h or with 5 µg/ml Tu for a course of 24 h and immunoblotted for the indicated proteins. **(D)** 5TGM1 cells were treated with 5 mM DTT for 3 h or with Tu for 24 h using increasing concentrations and immunoblotted for the indicated proteins. **(E)** 5TGM1 cells were treated with 5 mM DTT for 3 h or with 3.5 µM BFA for a course of 24 h and immunoblotted for the indicated proteins. **(F)** 5TGM1 cells were treated with 5 mM DTT for 3 h or with BFA for 12 h using increasing concentrations and were immunoblotted for the indicated proteins. **(G)** 5TGM1 cells were treated with 5 mM DTT for 3 h or with 50 µM MG132 for a course of 24 h and were immunoblotted for the indicated proteins. **(H)** 5TGM1 cells were treated with 5 mM DTT for 3 h or with MG132 for 4 h using increasing concentrations and were immunoblotted for the indicated proteins. **(I)** 5TGM1 cells were untreated or treated with 50 µM MG132 in the presence of 20 µM NH_4Cl, 20 µM chloroquine, or 20 µM Z-VAD-FMK for 12 h. Lysates were immunoblotted for the indicated proteins. **(J)** 5TGM1 cells were untreated or treated with 1 nM SubAB or 5 µg/ml Tu for 12 h, lysed, and immunoprecipitated for IRE1. Bead-bound IRE1 was treated with CIP for 3 h and immunoblotted for IRE1. **(K)** 5TGM1 cells were untreated or treated with 3.5 µM BFA for 12 h, lysed, and immunoprecipitated for IRE1. Bead-bound IRE1 was treated with CIP for 3 h and immunoblotted for IRE1. Data in this figure are representative of three independent experiments.

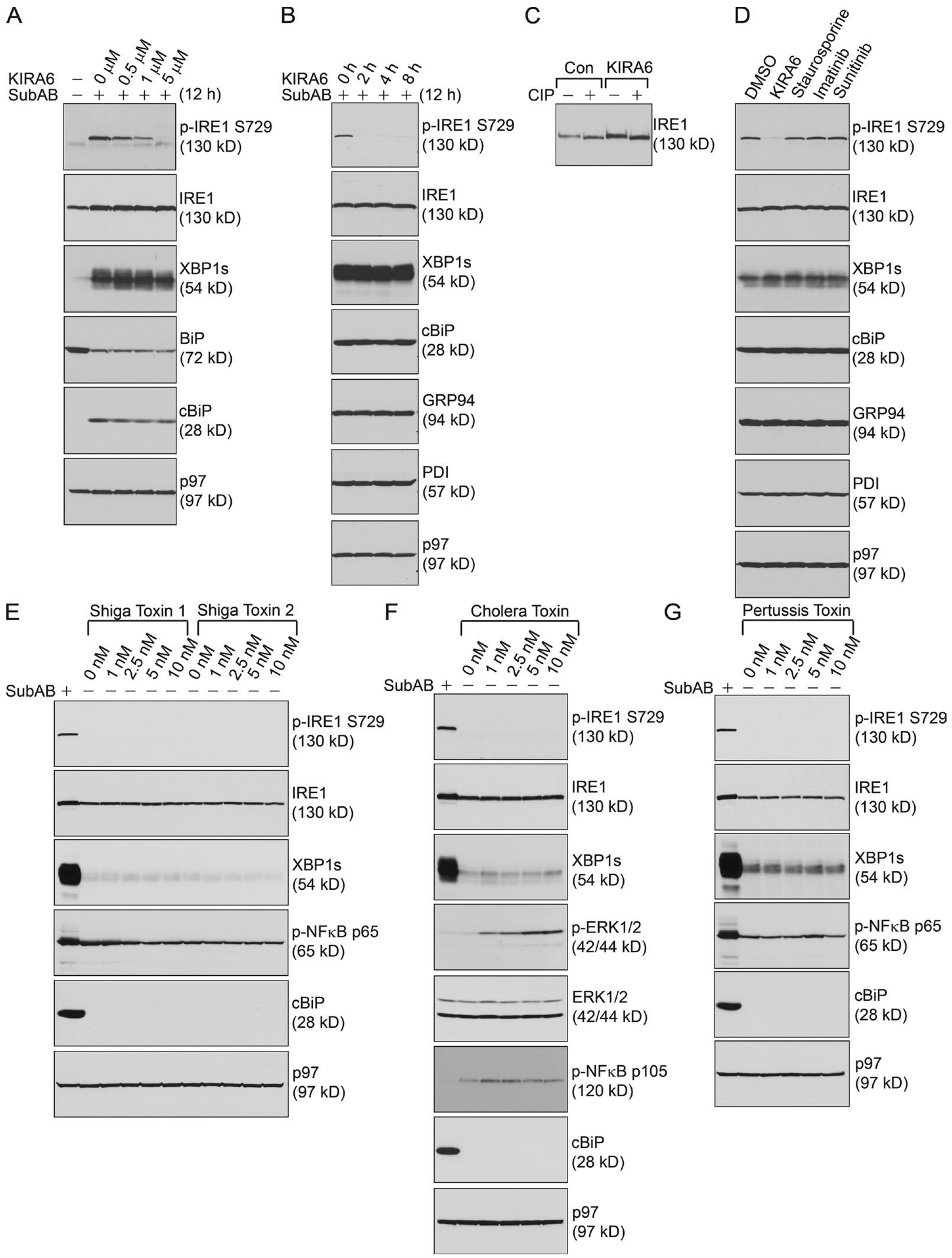

Figure S2. **KIRA6 rapidly suppresses SubAB-induced S729 phosphorylation of IRE1, and Shiga toxins, cholera toxin, and pertussis toxin do not induce S729 phosphorylation, even at high concentrations. (A)** 5TGM1 cells were treated with 1 nM SubAB in the presence of KIRA6 at the indicated concentrations for 12 h. Lysates were immunoblotted for the indicated proteins. **(B)** 5TGM1 cells were exposed to 1 nM SubAB for 12 h and were subsequently treated with 5 µM KIRA6 for a course of 8 h and immunoblotted for the indicated proteins. **(C)** 5TGM1 cells were untreated or treated with 5 µM KIRA6 for 12 h, lysed, and immunoprecipitated for IRE1. Bead-bound IRE1 was treated with CIP for 3 h and immunoblotted for IRE1. **(D)** 5TGM1 cells were exposed to 1 nM SubAB for 12 h and were subsequently treated with KIRA6, staurosporine, imatinib, or sunitinib at 5 µM for an additional 8 h and then immunoblotted for the indicated proteins. **(E)** 5TGM1 cells were treated with 1 nM SubAB for 24 h or with Shiga toxins 1 and 2 for 24 h using increasing concentrations and were immunoblotted for the indicated proteins. **(F)** 5TGM1 cells were treated with 1 nM SubAB for 24 h or with cholera toxin for 24 h using increasing concentrations and then were immunoblotted for the indicated proteins. **(G)** 5TGM1 cells were treated with 1 nM SubAB for 24 h or with pertussis toxin for 24 h using increasing concentrations and were immunoblotted for the indicated proteins. Data in this figure are representative of three independent experiments.

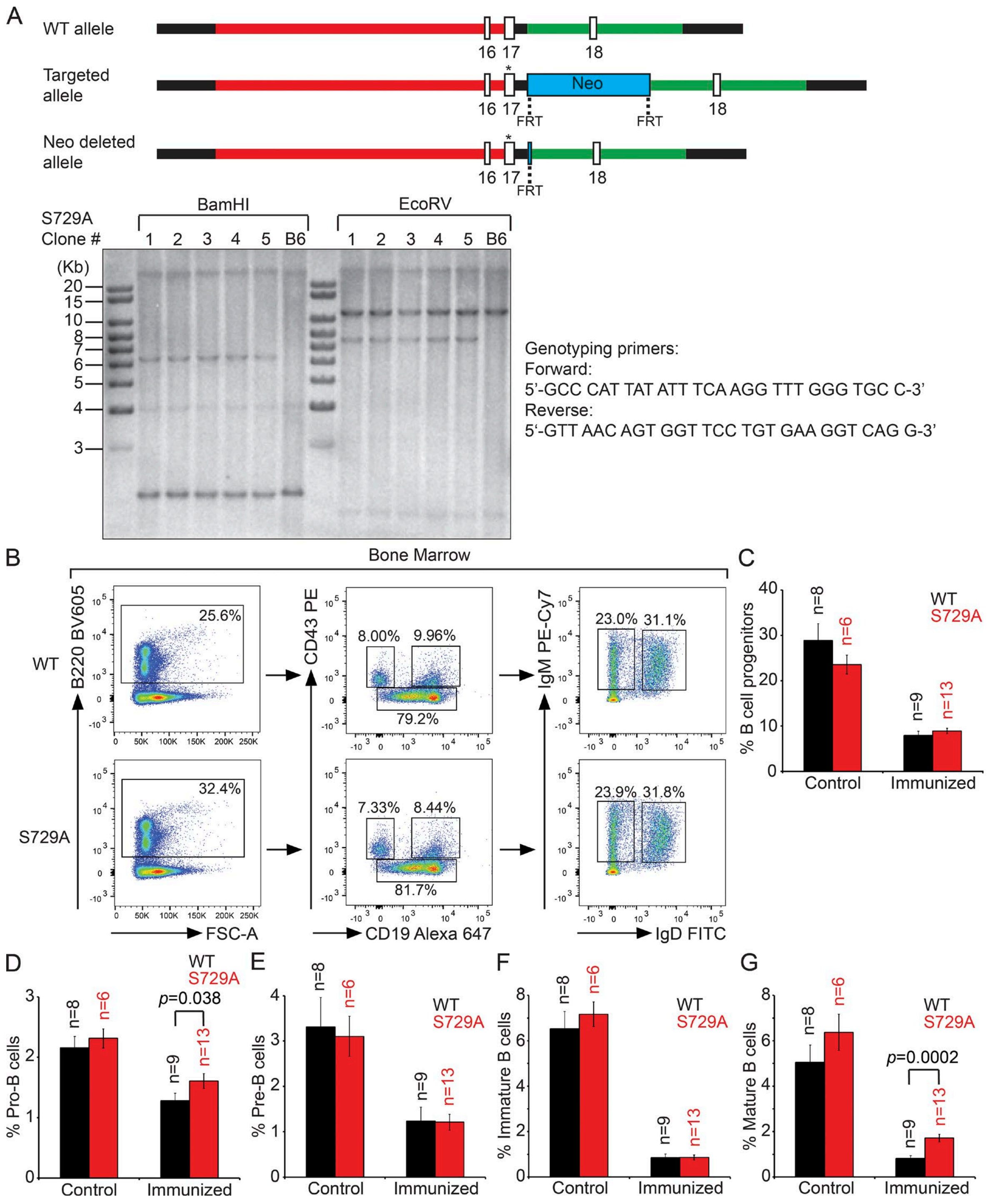

Figure S3. **Generation of the S729A mouse model. (A–G)** B cells develop normally in the bone marrow of unimmunized S729A mice; after immunization, the percentages of pro– and mature B cells increase in the bone marrow of S729A mice. **(A)** The S729 residue of mouse IRE1 is located on exon 17. WT, targeted, and Neo-deleted alleles are shown. S729A is shown with an asterisk. Genomic DNA was extracted from five different clones carrying the targeted allele and was digested with BamHI or EcoRV. Southern blots were performed with a radiolabeled probe to confirm the integration of 3′ and 5′ homology arms. The expected band sizes of the WT and targeted alleles after restriction digestion by BamHI were 2.1 kb and 6,332 bp, respectively. The expected band sizes of the WT and the targeted alleles after restriction digestion by EcoRV were 11.2 kb and 7,738 bp, respectively. Genomic DNA from WT B6 mice was used as a control. In addition, the sequences of primers used to genotype mice carrying the Neo-deleted allele carrying S729A are listed (A). **(B)** WT and S729A mice were unimmunized or immunized with HEL plus CFA and boosted three times with HEL plus IFA. Bone marrow cells isolated from WT and S729A mice were stained with B220-BV605, CD43-PE, CD19–Alexa Fluor 647, IgM-PE-Cy7, and IgD-FITC. Gated $B220^+$ B cell populations were analyzed for $CD43^+$/$CD19^{low}$ pro–B cells and $CD43^+$/$CD19^{high}$ pre–B cells (middle). The gated CD43-/$CD19^+$ population (middle) were also analyzed for IgM^+/IgD^- immature B cells and IgM^+/IgD^+ mature B cells (right). Data in B are representative flow cytometry plots, which were quantified as pooled data shown in C–G. **(C–G)** Quantifications of B cell progenitors (C), pro–B cells (D), pre–B cells (E), immature B cells (F), and mature B cells (G) in the bone marrow of multiple unimmunized and immunized WT and S729A mice are shown as means ± SEM.

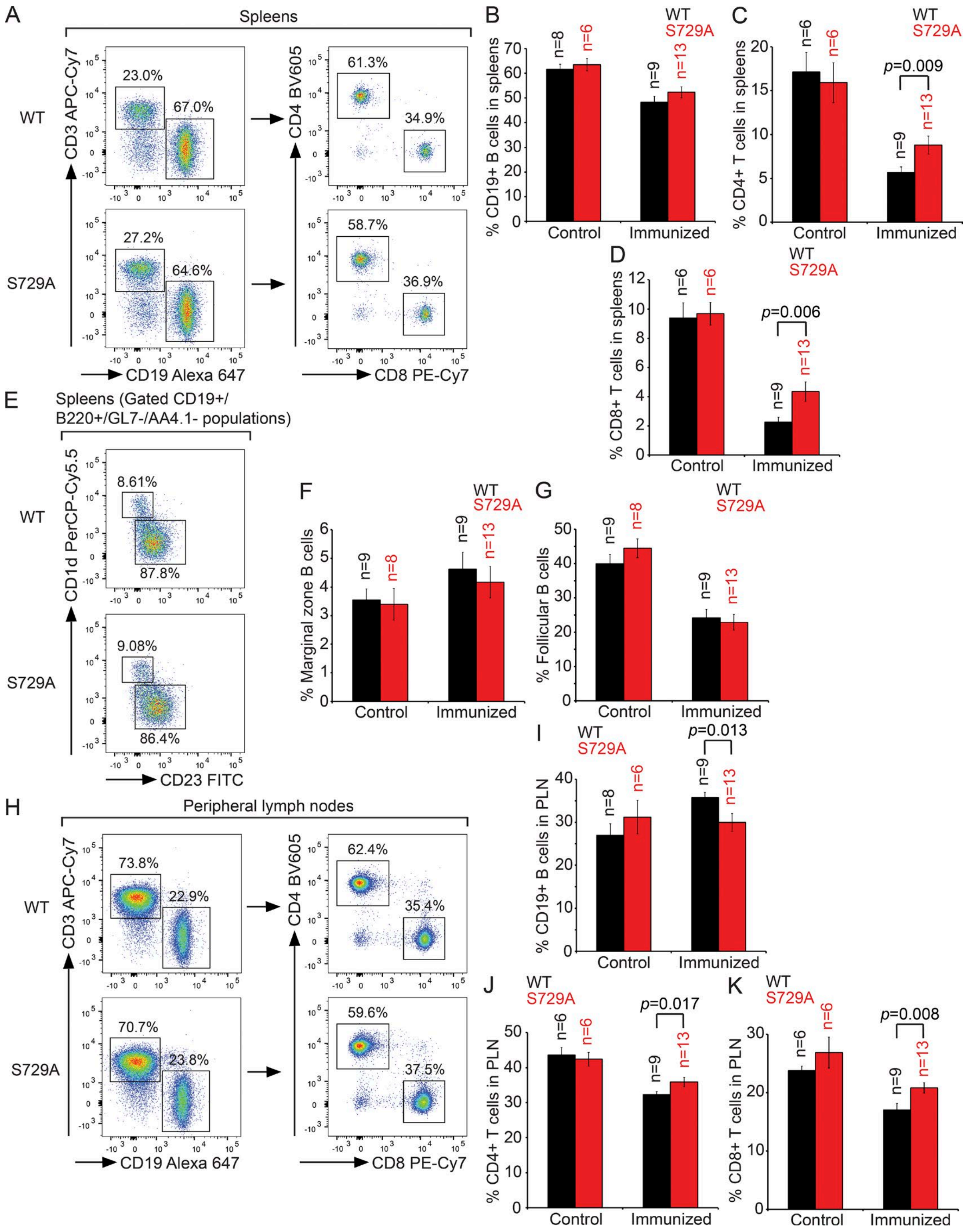

Figure S4. **Normal B and T cell percentages in the spleens and lymph nodes of unimmunized S729A mice; after immunization, the percentages of CD19⁺ B cells decrease in the lymph nodes, whereas the percentages of CD4⁺ and CD8⁺ T cells increase in the spleens and lymph nodes of S729A mice. (A–K)** WT and S729A mice were unimmunized or immunized with HEL plus CFA and boosted three times with HEL plus IFA. **(A)** Splenocytes from WT and S729A mice were stained with CD3-APC-Cy7, CD19–Alexa Fluor 647, CD4-BV605, and CD8-PE-Cy7. $CD3^+/CD19^-$ T cell populations were analyzed for $CD4^+$ and $CD8^+$ T cells. Data in A are representative flow cytometry plots, which were quantified as pooled data shown in B–D. **(B–D)** Quantifications of $CD19^+$ B cells (B), $CD4^+$ T cells (C), and $CD8^+$ T cells (D) in the spleens of multiple unimmunized and immunized WT and S729A mice are shown. **(E)** In addition, splenocytes from WT and S729A mice were stained with CD19–Alexa Fluor 647, B220-BV605, GL7-PE, AA4.1-PE-Cy7, CD1d-PerCP-Cy5.5, and CD23-FITC. Gated $CD19^+/B220^+/GL7^-/AA4.1^-$ B cell populations were analyzed for the $CD1d^+/CD23^-$ marginal zone and $CD1d^-/CD23^+$ follicular B cells. Data in E are representative flow cytometry plots, which were quantified as pooled data shown in F and G. **(F and G)** Quantifications of marginal zone (F) and follicular (G) B cells in the spleens of multiple unimmunized and immunized WT and S729A mice are shown. **(H)** Peripheral lymph node cells from WT and S729A mice were stained with CD3-APC-Cy7, CD19–Alexa Fluor 647, CD4-BV605, and CD8-PE-Cy7. $CD3^+/CD19^-$ T cell populations were analyzed for $CD4^+$ and $CD8^+$ T cells. Data in H are representative flow cytometry plots, which were quantified as pooled data shown in I–K. **(I–K)** Quantifications of $CD19^+$ B cells (I), $CD4^+$ T cells (J), and $CD8^+$ T cells (K) in the peripheral lymph nodes (PLNs) of multiple unimmunized and immunized WT and S729A mice are shown. Data are shown as means ± SEM.

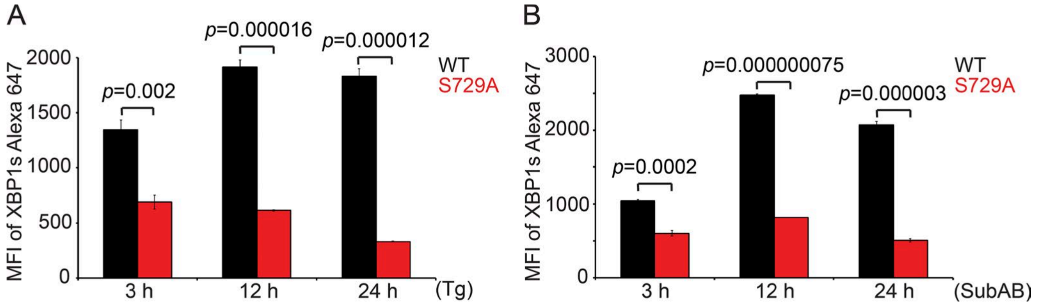

Figure S5. **LPS-stimulated B cells from S729A mice fail to respond to Tg or SubAB by enhancing the expression of XBP1s. (A and B)** Naive B cells purified from WT (*n* = 3) and S729A (*n* = 3) mice were stimulated with LPS for 2 d and exposed to 2.5 µM Tg (A) or 1.5 nM native SubAB (B) for an additional 3, 12, and 24 h. At each time point, B cells were stained with XBP1s–Alexa Fluor 647 and B220–Alexa Fluor 488. The gated B220$^+$ populations were analyzed for expression of XBP1s shown as MFI. Data are shown as means ± SD. The MFI data are representative of three independent experiments.
